# Testing the limits of gradient sensing

**DOI:** 10.1371/journal.pcbi.1005386

**Published:** 2017-02-16

**Authors:** Vinal Lakhani, Timothy C. Elston

**Affiliations:** 1 Curriculum in Bioinformatics and Computational Biology, University of North Carolina at Chapel Hill, Chapel Hill, North Carolina, United States of America; 2 Battelle Center for Mathematical Medicine, Research Institute at Nationwide Children’s Hospital, Columbus, Ohio, United States of America; 3 Department of Pharmacology, University of North Carolina at Chapel Hill, Chapel Hill, North Carolina, United States of America; University of California Irvine, UNITED STATES

## Abstract

The ability to detect a chemical gradient is fundamental to many cellular processes. In multicellular organisms gradient sensing plays an important role in many physiological processes such as wound healing and development. Unicellular organisms use gradient sensing to move (chemotaxis) or grow (chemotropism) towards a favorable environment. Some cells are capable of detecting extremely shallow gradients, even in the presence of significant molecular-level noise. For example, yeast have been reported to detect pheromone gradients as shallow as 0.1 nM/μm. Noise reduction mechanisms, such as time-averaging and the internalization of pheromone molecules, have been proposed to explain how yeast cells filter fluctuations and detect shallow gradients. Here, we use a Particle-Based Reaction-Diffusion model of ligand-receptor dynamics to test the effectiveness of these mechanisms and to determine the limits of gradient sensing. In particular, we develop novel simulation methods for establishing chemical gradients that not only allow us to study gradient sensing under steady-state conditions, but also take into account transient effects as the gradient forms. Based on reported measurements of reaction rates, our results indicate neither time-averaging nor receptor endocytosis significantly improves the cell’s accuracy in detecting gradients over time scales associated with the initiation of polarized growth. Additionally, our results demonstrate the physical barrier of the cell membrane sharpens chemical gradients across the cell. While our studies are motivated by the mating response of yeast, we believe our results and simulation methods will find applications in many different contexts.

## Author summary

In order to survive, many organisms must not only be able to detect the presence of a chemical compound, but also in which direction that compound increases or decreases in concentration. For example, bacteria cells prefer to move towards areas with high sugar concentrations. The process by which cells determine the direction of a chemical gradient is called “Gradient Sensing”. Of particular interest is the gradient sensing capability of yeast cells. These cells have been observed detecting the direction of extremely shallow gradients, which produce only a 2% difference in the number of molecules across the cell. Because the molecular-level noise is much larger than this signal, it is unclear what noise-reduction mechanism the cell employs to reduce the noise and detect the signal. We developed a 3D computational simulation platform to calculate and study the exact positions of molecules during this process. Our platform utilizes High Performance Computing clusters and GPGPUs. We find that, of the two prevailing models in the literature, neither time-averaging nor receptor endocytosis sufficiently reduces molecular noise for yeast cells to reliably detect chemical gradients before they initiate polarized growth. This finding implies yeast must possess a mechanism for reorienting the direction of growth after cell polarization has occurred. We also find the cell membrane and similarly, any other physical barrier nearby the cell can improve the cell’s likelihood of detecting the gradient. Our simulation methods and results will be applicable in other areas of research.

## Introduction

**Fig 1:**
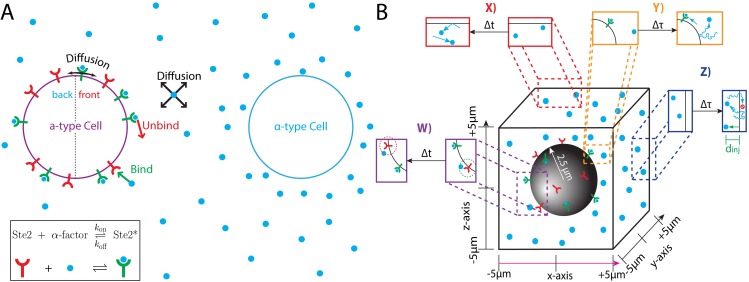
Computational platform for studying yeast gradient sensing. **(A)** *MAT*α cells emit the mating pheromone 'α-factor' (blue circle). Nearby *MAT***a** cells detect pheromone using the G-Protein Coupled Receptor Ste2 (red-shape = 'unbound' state and green-shape with blue circle = 'bound' state). Pheromone gradients must be detected in the presence of stochastic effects from pheromone binding (green arrow), unbinding (red arrow) and pheromone and receptor diffusion (black arrows). **(B)** Illustration of our Particle-Based Stochastic Reaction-Diffusion Model. During each time step (Δt), free receptors can capture pheromone molecules located within a specified capture radius (dashed green circle). Bound receptors randomly release a pheromone molecule a fixed distance (unbinding radius: dashed red circle) from the receptor. Pheromone molecules undergo 3D diffusion (each time step Δt) and receptors diffuse on the cell surface (with a coarser time scale Δτ). The ‘y’ and ‘z’ boundaries of the computational domain are reflective as well as the cell surface. Near the ‘x’ boundaries, pheromone molecules diffuse freely for a time (Δτ). After Δτ time, all pheromone molecules located outside the ‘x’ boundaries are removed. New pheromone molecules are injected every Δτ time near the ‘x’ boundaries; each new molecule is placed a random distance (‘injection distance’) away from the ‘x’ boundary (green arrow, d_inj_).

The ability to detect the direction of a chemical gradient is fundamental to many biological processes. To survive or carryout their proper function, individual cells must be able to undergo directed growth (chemotropism) or movement (chemotaxis) toward chemical signals, such as nutrients or hormones. An ideal system for studying gradient sensing is chemotropism during the mating response of *S*. *Cerevisiae* (yeast). Yeast cells can exist as one of two haploid types: *MAT***a** or *MAT*α. *MAT***a** cells seek a mating partner by sensing a gradient of the pheromone α-factor secreted by *MAT*α cells (**Fig 1A**).

Gradient sensing strategies fall into two major categories: temporal and spatial. Temporal sensing mechanisms, in which an organism moving through its environment compares the concentration between its current and previous locations, are commonly utilized by small cells such as *E*. *Coli* (~1μm). Spatial sensing mechanisms, in which the organism compares the concentration difference across the cell body, are commonly used by large cells including most eukaryotes, such as *D*. *Discoideum* (~15μm). The fact that yeast cells are not motile suggest they use a spatial sensing mechanism, despite being smaller (~4μm in diameter) than most eukaryotic cells.

Experimental studies have reported that yeast cells are capable of sensing linear gradients as shallow as 0.1 nM/μm [1,2]. All information on the extracellular pheromone gradient comes from receptors on the cell’s surface. Therefore, these receptors set the ultimate limits on gradient sensing. To quantify the challenges faced by a cell in detecting shallow gradients, we can estimate the average number of ligand-bound or active receptors (receptor occupancy) in the front half of the cell (pointing up the gradient) versus the back half of the cell (pointing down the gradient). We begin with estimating the size of the fluctuations about the mean receptor occupancy. The average receptor occupancy is roughly given by $n=N\frac{c}{K_{D}+c}$, where N is the total number of receptors; K_D_ is the dissociation constant, and c is the average concentration. The average occupancy in each half can be estimated using the average concentration in the front (or back) of the cell. This expression is an approximation because it does not correctly take into account the spatial dependence of the gradient across the cell. For a cell with 10000 receptors in a linear pheromone gradient of 0.1 nM/μm centered at the K_D_ (about 7 nM) of the receptor, the difference in receptor occupancy is Δn = n_front_−n_back_ ≈ 45. This calculation estimates a less than 1% difference in receptor occupancy between the front & back of the cell. Following the work of Lauffenburger [3], the magnitude of the fluctuations in receptor occupancy is $\sigma_{n}=\sqrt{N\frac{K_{D}∙c}{{\left(K_{D}+c\right)}^{2}}}\approx 50$. Hence for a gradient of 0.1 nM/μm, the signal is masked by the noise: Δn ≈ 45 ± 50, and a cell cannot predict the direction of the gradient based on an instantaneous measurement of receptor occupancy.

To explain how cells overcome these fluctuations, various noise-reduction mechanisms have been proposed: including time-averaging, gradient sharpening via extracellular degradation of pheromone by the protease Bar1 [4,5] and removal of active receptors via endocytosis to avoid resampling [6,7]. The limits of these mechanisms have been estimated using mathematical models. Time-averaging requires sufficient time for the cell to sample multiple binding and unbinding events. In the yeast mating system, the K_D_ of Ste2 binding to α-factor is known to be around 7nM [8–12]. Reported values for the unbinding rate are extremely slow: on the order of 10^−4^–10^−3^ s^-1^ [12,13]. These values imply a binding rate on the order of 10^4^–10^5^ (M∙s)^-1^, which is many orders of magnitude slower than the diffusion limit of approximately 10^9^ (M·s)^-1^. With a dissociation rate of 0.0011 s^-1^ [12], changes in receptor occupancy occur on the order of 10’s of minutes. Given that yeast cells begin chemotropic growth within 30 minutes of exposure to pheromone, there seems to be insufficient time for the cell to accurately sense a shallow gradient using the time-averaging mechanism alone. *MAT***a** cells secrete Bar1: a protease that degrades extracellular α-factor. This process is known to locally sharpen the pheromone gradients between neighboring cells and has been suggested as a mechanism for sensing shallow gradients and ensuring that two or more cells avoid competing for the same mate [4,5]. It is not known if this sharpening effect sufficiently reduces the noise to enable the cells to gradient sense. Active receptors are removed from the membrane through endocytosis and newly synthesized receptors are brought to the membrane on vesicles. This receptor cycling has been suggested as a mechanism to improve gradient sensing [6,7] by removing pheromone from the environment and preventing re-sampling of the same ligand molecule. Additionally, the endocytosis rate of active receptors, 0.0021 s^-1^ [14,15], is faster than reported unbinding rates. Therefore, receptor endocytosis may improve the sampling frequency.

The results discussed above are based on mathematical models in which simplifying assumptions are made to allow analytic tractability. To go beyond these models, we built a simulation platform based on fundamental biophysical processes that allows us to evaluate noise-reduction mechanisms and study gradient sensing with minimal assumptions. In particular, we develop a Particle-Based Stochastic Reaction-Diffusion Model to study receptor dynamics in a gradient (Fig 1B). We choose this approach, because non-particle-based methods using Reaction Diffusion Master Equations (RDME), although computationally fast, discretize space. This discretization implicitly assumes each computational voxel is well-mixed, which reduces spatial accuracy [16]. Additionally, these methods are difficult to implement when the computational domain has complex boundary conditions such as the partially absorbing boundaries we use to create a gradient. Currently available particle-based simulation packages, such as Smoldyn or MCell, treat 2^nd^ order reactions by assuming that once two reactants are within a specified capture radius, the reactions occurs with certainty [4,17]. However, for our system, the slow association rate would make the capture radius unphysically small. Thus, we choose to create our own Particle-Based Stochastic Reaction-Diffusion Model and use the methods developed by Erban and co-workers [18] to treat 2^nd^ order reactions. Their method allows for customization of the binding radius. We also develop novel methods for simulating the development of chemical gradients that do not exist in current publically available software packages.

Our simulations reveal: 1) time-averaging and receptor cycling, wherein Ste2 exocytosis is isotropic (unpolarized), are insufficient for yeast cells to confidently detect the direction of shallow gradients before initiating polarized growth, and 2) isotropic exocytosis of Bar1 may improve gradient sensing for cells with fast reaction rates. Additionally, our simulations reveal that 1) the physical barrier of the cell membrane sharpens the gradient, and 2) diffusion of the receptor reduces the cell’s ability to detect the direction of the gradient. Our approach bridges the theoretical mathematical models and *in vivo* experimental approaches.

## Results

We are motivated by the ability of yeast cells to sense shallow pheromone gradients even in the presence of significant amounts of molecular noise. Accordingly, we set the biophysical parameter values in our simulations to match the yeast mating response system (**Table 1**). Although the K_D_ of the Ste2 receptor is known to be around 7nM [8–12], there is no consensus for the binding and unbinding rates. The diffusion limit for binding is on the order of 10^9^ (M·s)^-1^. However, one experimental study reported rates as slow as k_on_ = 1.6×10^5^ (M·s)^-1^ and k_off_ = 0.0011 s^-1^ [12], and similar values were measured in [8]. Elsewhere, a computational model used rates ten times faster than these values; although, the experimental sources for these rates are unclear [11]. To compare how these rates affect gradient sensing, we consider both sets of reaction rates.

**Table 1:** Standard Parameter Set.

Free Parameters	Value	Description
XDom	10 μm	Length of x-domain
YDom	10 μm	Length of y-domain
ZDom	10 μm	Length of z-domain
Δt	1 μs	Time Step
Δτ	50 μs	Coarse Time Step
R	2.5 μm	Radius of Cell
D_α_	125 μm^2^/s	Pheromone Diffusion Constant [5]
Grad	0.1 nM/μm	Pheromone Concentration Gradient along x-axis
Conc	6.9 nM	Background Pheromone Concentration (equal to K_D_)
D_Ste2_	0.0025 μm^2^/s	Receptor Diffusion Constant [34]
N	10000	Number of Receptors on Cell Surface [8–11]
k_on_	1.6×10^5^ (M·s)^-1^	Binding Rate [12]
k_off_	0.0011 s^-1^	Unbinding Rate [12]
r_bind_	4 nm	Binding Radius
r_unbind_	4 nm	Unbinding Radius
**Computed Parameters**	**Value**	**Calculated from the above free parameters**
P_bind_	0.002	Binding Probability
P_unbind_	1.1×10^−9^	Unbinding Probability
K_D_	6.9 nM	Pheromone/Receptor Dissociation Constant [12]
c_high_	7.4 nM	Concentration at High Boundary (*x* = 5μm)
c_low_	6.4 nM	Concentration at Low Boundary (*x* = –5μm)
n_inj High_	19.88	Average Number of Pheromone to create at High Boundary
n_inj Low_	17.19	Average Number of Pheromone to create at Low Boundary
d_inj_	Random from Eq 15	List of injection distances: random numbers from Eq 15

The first set of values contains all the parameters necessary to uniquely define a stochastic simulation. The second set contains additional values that are calculated from the first set of parameters.

In Section I, we simulate a cell whose receptors are in equilibrium with a uniform pheromone concentration. From these simulations, we quantify noise levels and investigate noise-reduction through time-averaging. We compare our simulation results to previously published theoretical studies. In Section II we simulate a cell whose receptors are in equilibrium with a pheromone gradient. We report on how the cell’s presence in the gradient generates non-linear effects on the pheromone concentration and the contribution of receptor diffusion to fluctuations in the distribution of active receptors. In Section III we simulate a cell experiencing the formation of a pheromone gradient and report on the role of the protease Bar1 on the cell’s ability to sense gradients.

### Section I: Equilibrium fluctuations in uniform pheromone concentration

#### Equilibrium fluctuations in receptor occupancy

Since the seminal work of Berg & Purcell [19], there have been many theoretical studies on the limits with which cells can measure external ligand concentrations [6,20–23] and strategies for overcoming these limitations [6]. The two main sources of fluctuations considered by these studies are fluctuations in the ligand concentration and stochastic binding and unbinding of the ligand from the receptor. Our simulations accurately capture these sources of noise as well as fluctuations from receptor diffusion. Our model also allows us to investigate non-equilibrium conditions and the effect of the cell in perturbing the ligand concentration profile. We show our simulation results agree most closely with the theory of Berezhkovskii and Szabo [20].

**Fig 2:**
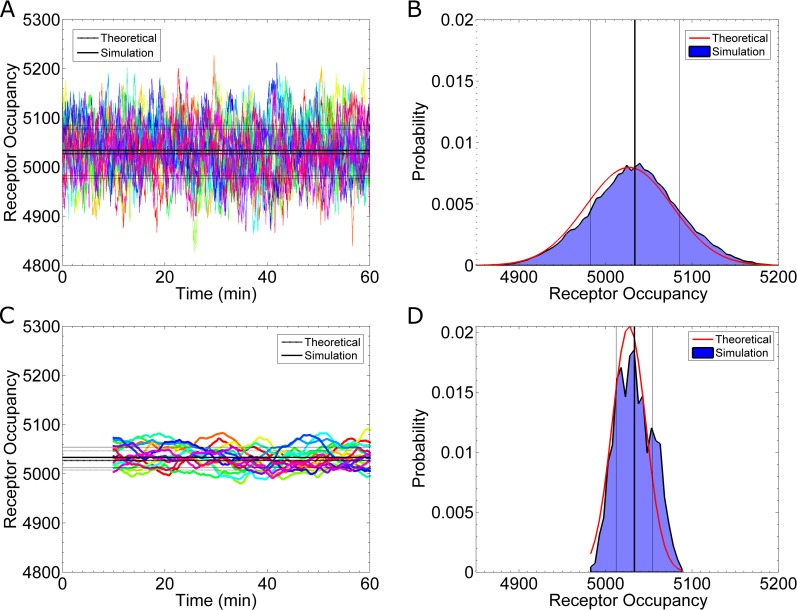
Receptor Occupancy at Equilibrium. **(A)** Simulation results for the number of active receptors as a function of time. Each color represents a different realization of the process. The thick, solid, black line is the mean from the data (5035 Ste2*). The thin, solid, black lines represent one standard deviation away from the mean, as calculated from the data (±52 Ste2*). The thick, dashed, black line is the theoretical mean calculated from Eq 1a (5027 Ste2*). The thin, dashed, black lines are one theoretical standard deviation from the mean as calculated from Eq 1b (±50 Ste2*). **(B)** A histogram of the data in **(A)**. The vertical lines are equivalent to those in **(A)**. The red curve shows the theoretical distribution. **(C)** A plot of time-averaged receptor occupancy. Each time point displays the average occupancy of the preceding 10 minutes. No average is available for t < 10min. The black lines are similar to those in **(A)**. The simulation mean is 5034 ± 23 Ste2*, and the theoretical mean is 5027 ± 19 Ste2*. The theoretical mean is again calculated with Eq 1a, but the time-averaged standard deviation is calculated from Eq 5 and the relation described in the text. **(D)** A histogram of the data in **(C)**. The time-averaged distribution is much narrower (σ = 23 Ste2*) than the instantaneous distribution (σ = 52 Ste2*). The parameters used for these simulations are reported in **Table 1**, with binding and unbinding rates of 1.6×10^6^ (M·s)^-1^ and 0.011 s^-1^, respectively, and no ligand gradient.

Most models assume the cell’s receptors are at equilibrium with an external, uniform ligand concentration. Therefore, we begin by performing simulations under equilibrium conditions. First, we initialize the system to have the expected number of active receptors and ligand molecules in the computational domain. Second, we allow the system to equilibrate for 30-minutes to generate a random state. Lastly, this random state is used as the initial condition for a 60-minute simulation, which we then analyze. **Fig 2**shows the results of sixteen simulations using the parameters listed in **Table 1**, with the exceptions that: 1) there is no ligand gradient (*g* = 0 nM/μm) and 2) the reaction rates are taken to be k_on_ = 1.6×10^6^ (M·s)^-1^ and k_off_ = 0.011 s^-1^. **Fig 2A** shows the receptor occupancy time series, n(t), and **Fig 2B** shows a histogram of the data.

For uniform pheromone concentration, the mean and standard deviation can be calculated from the following equations:

$$\bar{n}=N\frac{c}{K_{D}+c}$$ (1a)

$$\sigma_{n}=\sqrt{N\frac{K_{D}\;c}{{\left(K_{D}+c\right)}^{2}}}$$ (1b)

(derived in [3]). In both equations, *N* is the total number of receptors; K_D_ is the dissociation constant, and *c* is the concentration. In Eq 1a, $\bar{n}$ is the average receptor occupancy, and in Eq 1b, σ_n_^2^ is the variance of the receptor occupancy. Our simulations match the mean and standard deviation of the receptor occupancy with the theoretical values (**Fig 2A and 2B**).

#### Time averaging

A common noise reduction technique from signal-processing is time-averaging. Assume the receptor occupancy is averaged for a length of time, *T*, then starting with the instantaneous occupancy n(t) (**Fig 2A**), we calculate the time average as $n_{T}(t)=\frac{1}{T}\sum_{i=1}^{T/\Delta t}\;n(t-T+\Delta t∙i)$, where Δt is sampling interval. **Fig 2C** shows the time-averaged occupancy for T = 10min. The resulting time-averaged standard deviation, which we label as $\sigma_{n_{T}}$, is 23 Ste2* molecules (**Fig 2C and 2D**). This time-averaged uncertainty is much smaller than the instantaneous uncertainty, which is 52 Ste2* molecules (**Fig 2A and 2B**). **Fig 2D** shows the corresponding time-averaged histogram. The theoretical time-averaged values (dashed lines in **Fig 2C** and red curve in **Fig 2D**) are calculated from the theoretical work of Berezhkovskii and Szabo [20]. Below, we discuss how these results compare with the other theoretical models that have appeared in the literature.

In 1977, Berg & Purcell derive an expression for the lower bound on the accuracy a cell can achieve when time-averaging [19]:

$${\text{CV}}^{2}=\frac{\sigma_{c}{}^{2}}{c^{2}}=\frac{1}{\pi DRcT}\left(1+\frac{k_{\text{on}}c}{k_{\text{off}}}\right)$$ (2)

where $\frac{{\sigma_{c}}^{2}}{c^{2}}$ is the time-averaged variance in the concentration estimation divided by the average concentration squared. Because this ratio is the Coefficient of Variation squared, we label it CV^2^. In Eq 2, *D* is the diffusion constant for the ligand; *R* is the radius of the cell; *c* is the concentration of ligand, and *T* is the length of time-averaging. The Berg & Purcell model assumes the binding rate is diffusion-limited and the cell has an excessive number of receptors ($N\approx \frac{DR}{k_{on}}$) [19]. Hence, their conclusion shows the cell’s accuracy is limited by the stochastic arrival of ligand molecules to the cell [19]. In 2005, Bialek & Setayeshgar derived the CV^2^ for a binding rate slower than the diffusion-limit [21]:

$${\text{CV}}^{2}=\frac{\sigma_{c}{}^{2}}{c^{2}}=\frac{2}{k_{\text{on}}cT}\left(1+\frac{k_{\text{on}}c}{k_{\text{off}}}\right)+\frac{1}{\pi DRcT}$$ (3)

Eqs 2 & 3 were reconciled in 2014 by Kaizu et. al. [22]:

$${\text{CV}}^{2}=\frac{\sigma_{c}{}^{2}}{c^{2}}=\frac{2}{k_{\text{on}}cT}\left(1+\frac{k_{\text{on}}c}{k_{\text{off}}}\right)+\frac{1}{2\pi DRcT}\left(1+\frac{k_{\text{on}}c}{k_{\text{off}}}\right)$$ (4)

The second term in Eqs 3 & 4 is the contribution from fluctuations in the arrival of ligand molecules, similar to Eq 2. The first term in Eqs 3 & 4 is the contribution from stochastic binding and unbinding reactions. Eqs 3 & 4 were derived for a single receptor in solution [24]. In 2013, Berezhkovskii & Szabo derived an expression for the CV^2^ that includes both major sources of noise and considers an arbitrary number of receptors [20]:

$${\text{CV}}^{2}=\frac{\sigma_{c}{}^{2}}{c^{2}}=\frac{2}{Nk_{\text{on}}cT}\left(1+\frac{k_{\text{on}}c}{k_{\text{off}}}\right)+\frac{1}{2\pi DRcT}\left(1+\frac{k_{\text{on}}c}{Nk_{\text{off}}}\right)$$ (5)

**Fig 3:**
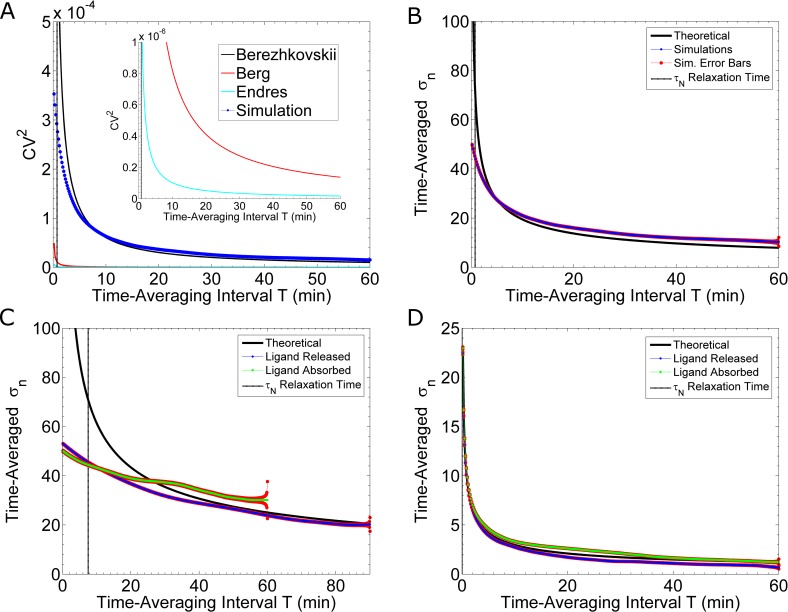
Noise Reduction by Time-Averaging. **(A)** Comparison of theoretical and simulation results. The black curve is a plot of Eq 5, taken from Berezhkovskii & Szabo [20]; the black, dashed, vertical line is the relaxation time, τ_N_. The theory is valid for T >> τ_N_ [20]. The red curve is a plot of Eq 2, taken from Berg & Purcell [19]. The teal curve is a plot of Eq 6, taken from Endres & Wingreen [6]. The blue curve is calculated from the simulations shown in **Fig 2A**. Inset: Zoom in along the y-axis. **(B)** As in **(A)**, the blue curve, with red standard error bars, is the result of simulations with fast reaction rates: 1.6×10^6^ (M·s)^-1^ and 0.011 s^-1^. The theoretical curve is from Eq 5. **(C)** Comparison of Ligand Releasing and Ligand Absorbing models. These simulations use the measured (see [12]) binding rate of k_on_ = 1.6×10^5^ (M·s)^-1^ and either an unbinding rate of k_off_ = 0.0011 s^-1^ (blue curve) or an endocytosis rate of k_Endo_ = 0.0011 s^-1^ (green curve). The blue curve is calculated from twenty-six simulations, and the green curve is calculated from eight simulations. The standard error for each calculation is shown in red. **(D)** Comparing the Ligand Releasing and Ligand Absorbing models. These simulations use rates 500 times faster than those in **(C)**. That is, the binding rate is k_on_ = 8×10^7^ (M·s)^-1^; the unbinding rate is k_off_ = 0.55 s^-1^ (blue curve); while, the endocytosis rate is k_Endo_ = 0.55 s^-1^ (green curve). We calculate the blue and green curves from ten simulations each. The standard error for each calculation is shown in red.

*N* is the total number of receptors on the cell surface. Note for *N* = 1, Eq 5 is equivalent to Eq 4. For comparison, we plot Eqs 2 & 5 along with the results from our simulations (**Fig 3A**). In order to compare our simulation data to these theoretical models, we must express our simulation data as CV^2^. From the receptor occupancy data in **Fig 2A**, we calculate time-averages of varying lengths (T = {10, 20 … 3600} sec). For each value of *T*, we compute the time average for each simulation (e.g. the case of T = 10min is shown in **Fig 2C**) and then use these results to calculate the time-averaged variance: ${\sigma_{n_{T}}}^{2}$. We also can calculate the instantaneous variance in receptor occupancy, σ_n_^2^ from the data presented in **Fig 2A**. Therefore, as described in [20], we calculate an empirical CV^2^ using the relationship: ${CV}^{2}=\frac{{\sigma_{n_{T}}}^{2}}{{\sigma_{n}}^{4}}$.

In **Fig 3A** we compare the theoretical results of the Berg & Purcell model [19] (red curve, Eq 2), the Berezhkovskii & Szabo model [20] (black curve, Eq 5) and our simulation results (dotted, blue curve). As discussed above, Eq 2 is derived from a model which predicts the cell’s accuracy is solely limited by the stochastic arrival of pheromone molecules [19]. Eq 5 is derived from a model which predicts the cell’s accuracy is additionally limited by the stochasticity of binding and unbinding reactions [20]. Our simulated data agree well with the results of Berezhkovskii & Szabo (**Fig 3A**), demonstrating that the stochastic binding and unbinding events contribute significantly to fluctuations in receptor occupancy.

A more intuitive representation of uncertainty is to plot the time-averaged standard deviation, $\sigma_{n_{T}}$, as a function of the length of time-averaging, *T*. We show that our simulations (blue curves, **Fig 3B–3D**) agree with the theoretical predictions from Berezhkovskii and Szabo [20] (black curves, **Fig 3B–3D**). In addition, results from our simulations are complementary to the theoretical model, in that, our simulations can estimate the uncertainty of short time-averaging lengths, for which the theory fails. Specifically, the theoretical model is valid only for T >> τ_N_, where, τ_N_ is the equilibration time scale of the system or ‘relaxation time’ [20]. In the case of slow reaction rates (k_on_ = 1.6×10^5^ (M·s)^-1^ and k_off_ = 0.0011 s^-1^), τ_N_ ≈ 7.5min, which is roughly the inverse of the sum of the rates. The theoretical predictions diverge from our simulation results for T < 40min (blue curve, **Fig 3C**). This time scale is comparable to the time at which yeast cells exposed to pheromone initiate polarized growth (~ 30 minutes after pheromone exposure). Hence our simulated results estimate the time-averaged uncertainty for biologically relevant time-scales, which current theories do not capture. In **Fig 3B–3D**, we compare the effectiveness of time-averaging between the two different sets of reaction rates discussed above. For fast rates, as used in [11], there is significant noise-reduction when time-averaging for short lengths of time: 10min or less (**Fig 3B**). Whereas, for slow rates, as measured in [12], time-averaging for as long as 20min only nominally reduces the noise (blue curve, **Fig 3C**). Thus, accurate measurements of the reaction rates are critical for determining how effectively time averaging can reduce noise.

#### The “perfectly absorbing” cell and receptor cycling

In addition to time-averaging, Endres & Wingreen propose that a cell which “perfectly absorbs” ligand molecules, can more accurately measures an external ligand concentration [6]. A “perfect absorber” is a cell which, after binding a ligand molecule, does not release that ligand molecule back into the environment. Therefore, Endres & Wingreen argue, the cell does not count the same ligand molecule more than once. Similar to Berg & Purcell’s work [19], this model assumes that the binding rate is diffusion-limited and that there are an infinite number of receptors. Endres & Wingreen derive the following expression for the cell’s accuracy under this mechanism [6]:

$${\text{CV}}^{2}=\frac{\sigma_{c}{}^{2}}{c^{2}}=\frac{1}{4\pi DRcT}$$ (6)

We find this model does not match our simulation results (**Fig 3A**). We can analytically compare this model to the Berezhkovskii & Szabo model [20], which best matches our simulation data. Eq 5 is a more general expression for the CV^2^ and reduces to a form similar to Eq 6 under certain limiting conditions. If $N\gg \frac{c}{K_{D}}$, then the second term in Eq 5 simplifies: $\frac{1}{2\pi DRcT}\left(1+\frac{k_{on}c}{{N\;k}_{off}}\right)\approx \frac{1}{2\pi DRcT}$. If $k_{on}cN(1-\bar{n})=k_{on}cN/\left(1+\frac{k_{on}c}{k_{off}}\right)\gg 4\pi DRc$, where $\bar{n}$ is the expected fractional occupancy, then the first term in Eq 5 is much smaller than the second term, and the expression reduces to the following expression:

$${\text{CV}}^{2}=\frac{\sigma_{c}{}^{2}}{c^{2}}\approx \frac{1}{2\pi DRcT}$$ (7)

The two conditions used to derive the above expression, are closely related to the assumptions made for Eqs 2 & 6. The first condition, $N\gg \frac{c}{K_{D}}$, indicates the need for many receptors; for typical parameter sets, this condition is easily met. The second condition indicates that every ligand molecule which encounters the cell must be captured by an unbound receptor. This condition can be met by having a high reaction rate (k_on_), a large number of receptors (*N*), or both. Hence, if there are enough receptors and the binding rate is fast enough to guarantee the capture of every ligand molecule, then the cell’s accuracy is given by Eq 7 [20] (see also “The Perfect Instrument” in [19]).

The parameters in our simulations of the yeast system fail to meet these conditions and cannot be considered a “perfect absorber”. The binding rate is 3–4 orders of magnitude too slow. Nonetheless, we can simulate a partial absorber to determine if removing ligand from the environment, rather than releasing the ligand back into the environment, can improve the cell’s accuracy. A partial absorber does not absorb every ligand molecule that arrives at the surface. Endres & Wingreen suggest receptor cycling is a potential biological mechanism which enables the cell to absorb ligand [6]. Thus, we modify our simulation algorithm to include a simplified receptor cycling mechanism. In particular, we make the following changes to our algorithm (see Methods: “Receptor Cycling Model”). An active receptor, Ste2*, has some rate of endocytosis. Immediately upon endocytosis, an unbound receptor is created in a random position on the cell surface. Thus, in our simplified model, endocytosis is coupled with immediate replacement, which allows us to keep the total number of receptors constant. We also assume, Ste2* cannot unbind a ligand (k_off_ = 0 s^-1^). We find that absorbing ligand molecules does not reduce the fluctuations in time-averaged receptor occupancy as compared to unbinding and releasing ligand molecules (**Fig 3C and 3D**).

### Section II: At equilibrium in pheromone gradient

#### Gradient sharpening due to steric effects

**Fig 4:**
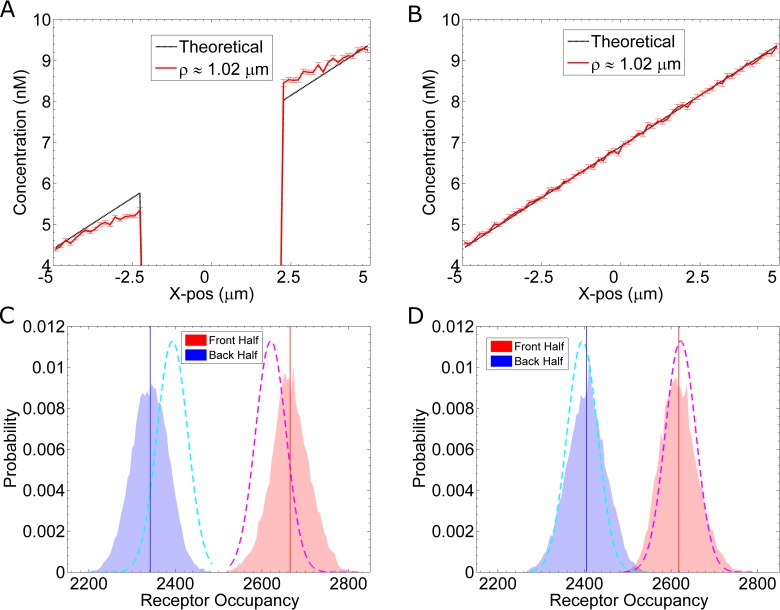
Gradient Sharpening Due to Steric Effects. We plot the pheromone concentration as a function of *x*, with $\rho =\sqrt{y^{2}+z^{2}}\approx 1.02\mu m$. The red curve is calculated from our simulations, which include binding and unbinding reactions. The dashed, black line is the ideal, linear concentration profile. **(A)** Results from eight simulations in which pheromone molecules reflect off the cell surface. There is no pheromone in the cell interior (x = –2.28μm to x = 2.28μm). **(B)** Same as **(A)**, except that the reflecting cell boundary has been removed. That is, pheromone molecules can diffuse through the cell membrane, but are still able to bind receptors. **(C)** Histograms from the simulation results shown in **(A)**. The blue and red histograms show the distributions for the number of active receptors, Ste2*, located in the back and front half of the cell, respectively. The solid vertical lines indicate the mean for their respective distributions. The dashed curves indicate the theoretical distribution for a cell in a linear gradient. **(D)** Same as **(C)** using data from simulations in **(B)**, that is, with a permeable cell membrane.

To establish a linear pheromone gradient in our simulations, we fix the pheromone concentration at the boundaries located at x = 5μm and x = –5μm (see Methods: “Gradient Method 1”). For example, to create a 0.5 nM/μm gradient with a concentration of 6.9nM at the midpoint, the concentration at x = –5μm is set to 4.4nM, and the concentration at x = 5μm is set to 9.4nM. In addition to these two boundaries, we set the boundary of the cell to be reflective, because the cell membrane is impermeable to pheromone. This impermeable boundary produces non-linear effects on the pheromone gradient, which we verify by calculating the concentration distribution within the computational space. For the calculation, we discretize the simulation space and count the average number of molecules in each bin. The molecules are tallied based on their x-position and distance from the x-axis ($\rho =\sqrt{y^{2}+z^{2}}$). Additional details on this calculation are provided in the Supplemental Methods (**S1 Text, Section E**). **Fig 4A** shows the average pheromone concentration profile for simulations (including reactions) with an impermeable cell membrane. This effect is not due to boundary conditions, because a similar profile is observed in simulations using larger volumes: (15 μm)^3^ (**S2 Text, Section A**). To confirm that the non-linear effects are solely due to the physical boundary (and not due to the biochemical reactions), we repeat the simulations but allow pheromone molecules to freely diffuse through the membrane. **Fig 4B** shows a profile for simulations (including reactions) with a permeable cell membrane. Without the diffusive barrier of the cell membrane, the concentration profile is linear (**Fig 4B**). Thus, we find the impermeability of the cell membrane produces nonlinear steric effects on the pheromone gradient, such that, the resulting gradient is steeper than expected (**Fig 4A and 4B**).

This sharpening effect is solely due to the impermeability of the cell membrane to pheromone. Simulations in which pheromone are free to diffuse through the cell membrane, produce a linear gradient: $g=\frac{\partial c}{\partial x}$ (**Fig 4B**). However, for an impermeable membrane, there is no flux across the cell boundary. Therefore, according to Fick’s Law, $J=-D\frac{\partial c}{\partial \hat{n}}=0$ or $\frac{\partial c}{\partial \hat{n}}=0$, where $\hat{n}$ is the vector normal to the cell surface. As predicted by this argument, the measured gradient $\frac{\partial c}{\partial x}$ near the boundary is close to 0 (**Fig 4A**). This constraint produces a higher than expected concentration at the front of the cell and a lower than expected concentration at the back of the cell. We next investigate whether this sharper gradient produces an appreciable difference in the distribution of active receptors.

We first consider the fast set of reaction rates: k_on_ = 1.6×10^6^ (M·s)^-1^ and k_off_ = 0.011 s^-1^. As an initial approach to measuring the distribution of Ste2*, we evaluate the receptor occupancy in the “front half” (x > 0μm) and the “back half” (x < 0μm). Assuming a true linear gradient, c(x) = gx + c_0_, we can calculate the mean and standard deviation of receptor occupancy using:

$$n=N\frac{1}{b-a}\int_{a}^{b}\frac{c\left(x\right)}{K_{D}+c\left(x\right)}\;dx$$ (8a)

$$\sigma_{n}{}^{2}=N\frac{1}{b-a}\int_{a}^{b}\frac{K_{D}\;c\left(x\right)}{{\left(K_{D}+c\left(x\right)\right)}^{2}}\;dx$$ (8b)

which are more generalized forms of Eq 1. For example, in the front half, a = 0 and b = R, which gives the following expression for the mean occupancy: ${\bar{n}}_{front}=N+N\frac{K_{D}}{gR}\ln\left(\frac{K_{D}+c_{0}}{K_{D}+c_{0}+gR}\right)$. Using Eq 8, we estimate that in a true linear gradient, the number of active receptors in the back half is n_back_ = 2395 ± 35 Ste2*, and the number of active receptors in the front half is n_front_ = 2622 ± 35 Ste2* (dashed lines, **Fig 4C and 4D**). From the simulation data, we count the number of occupied receptors located in each half at a given time. From simulations with an impermeable membrane, we calculate n_back_ = 2341 ± 42 Ste2* and n_front_ = 2665 ± 44 Ste2* (**Fig 4C**). From simulations, in which pheromone are allowed to pass through the cell membrane unimpeded, we calculate n_back_ = 2404 ± 45 Ste2* and n_front_ = 2617 ± 43 Ste2* (**Fig 4D**). Thus, we find steric effects from the cell membrane can locally sharpen the pheromone gradient. In turn, this sharpening can significantly improve the asymmetry in the distribution of active receptors (**Fig 4C and 4D**). For a 0.5 nM/μm gradient, the difference in active receptors between the front and back (Δn = n_front_−n_back_) changes from Δn = 212 Ste2* without sharpening to Δn = 324 Ste2* with sharpening, a more than 40% improvement.

#### Slow reaction rates and receptor diffusion add spatial noise

**Fig 5:**
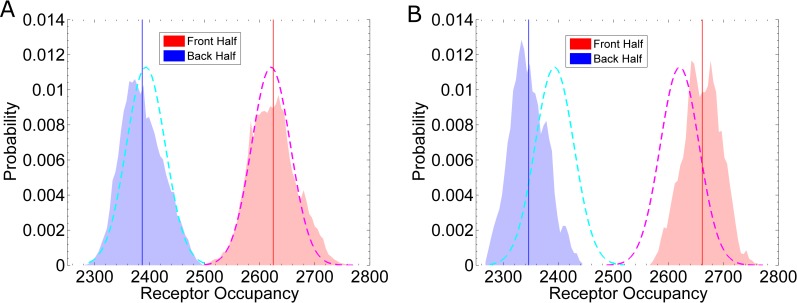
Receptor Diffusion adds Spatial Noise. These curves are similar to those in **Fig 4**except with slow reaction rates: k_on_ = 1.6×10^5^ (M·s)^-1^ and k_off_ = 0.0011 s^-1^. **(A)** Results from eight simulations. The difference in receptor occupancy is Δn = 238 Ste2*. **(B)** Results from eight simulations with slow reaction rates and no receptor diffusion; that is, D_Ste2_ = 0 μm^2^/s. The difference in receptor occupancy is Δn = 315 Ste2*.

As shown in **Fig 4**, the sharpened gradient increases the difference in receptor occupancy between the front and back for a system with fast reaction rates. To evaluate the sensitivity of the system with respect to the reaction rates, we repeat the simulations using the slow set of reaction rates: k_on_ = 1.6×10^5^ (M·s)^-1^ and k_off_ = 0.0011 s^-1^. We find that, although the pheromone gradient is equivalently sharpened (**S2 Text, Section B**), the simulations with slow reaction rates do not show a larger than expected difference in receptor occupancy between the front and back (**Fig 5**). Simulations with slow kinetics have a smaller difference in receptor occupancy, Δn = 238 Ste2* (**Fig 5A**), than simulations with fast kinetics: Δn = 324 Ste2* (**Fig 4C**). The cause for this discrepancy is receptor diffusion. A system with slow kinetics can increase the difference in receptor occupancy by reducing the receptor diffusion constant. For example, simulations with slow kinetics and no Ste2 diffusion (D_Ste2_ = 0 μm^2^/s) (**Fig 5B**) show a larger difference in receptor occupancy, Δn = 315 Ste2*, than simulations with receptor diffusion (**Fig 5A**).

One interpretation is that, a system with a slow unbinding rate allows activated receptors to diffuse long distances before reverting to the inactive state. For example, with a slow unbinding rate of k_off_ = 0.0011 s^-1^, a Ste2* molecule typically diffuses $\sqrt{2D_{Ste2}(\frac{1}{k_{off}})}=2.2\mu m$, or $\frac{2.2\mu m}{2.5\mu m}*\frac{180{}^{\circ}}{\pi }\approx 51{}^{\circ}$, away from the position at which it bound a pheromone molecule. Hence, many Ste2* molecules will diffuse from the front half to the back half of the cell or vice-versa. Because there are more Ste2* in the front half than back half, there is a net flux of Ste2*from the front to the back. This flux reduces Δn: the difference in receptor occupancy between the front and back halves. Sufficiently fast unbinding rates minimize this source of noise. Alternatively, endocytosis of active receptors would also act to minimize this effect. Gradient sensing could also be improved if there are two different receptor diffusion rates for free receptors and those which are ligand–bound. In *S*. *pombe*, for example, the pom1p protein has been shown to have two diffusion constants: a slow constant when the protein is part of a cluster and a fast constant otherwise [25]. This two-state model is sufficient for creating a pom1p gradient on the cell membrane [25]

#### Time-averaging the estimated gradient direction

**Fig 6:**
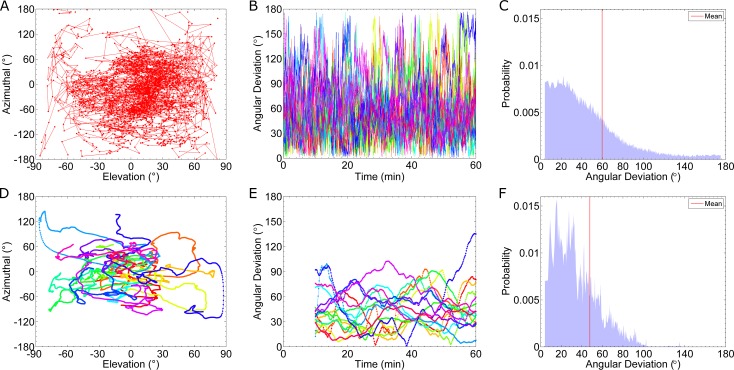
Estimated Angular Deviation from Gradient. Results from sixteen simulations of cells in a 0.1 nM/μm gradient, using the fast binding and unbinding rates: k_on_ = 1.6×10^6^ (M·s)^-1^ and k_off_ = 0.011 s^-1^. In this figure we plot the cell’s estimate of the gradient, *g*_*est*_, which is equal to the center of mass of the Ste2* distribution. “Elevation” measures the angle of *g*_*est*_ relative to the x-y plane, and “Azimuthal” measures the counterclockwise angle in the x-y plane away from the positive x-axis. The “Angular Deviation” is the angle between the true gradient and *g*_*est*_. **(A)** Trajectory in the azimuthal-elevation phase plane of a single simulation. **(B)** Plots of the instantaneous angular deviation from sixteen cells. Each color represents a single simulation result. **(C)** Histogram of the data in **(B)**. The average angle angular deviation is indicated by the vertical red line; the mean is 59.7°. **(D)** Time-averaged (10 min) trajectories in the azimuthal-elevation phase plane of sixteen simulations. **(E)** Plots of the time-averaged angular deviation (10 min). **(F)** Histogram of the data in **(E)**. The average angular deviation is indicated by the vertical red line; the mean is 47.8°.

Up to this point, we have limited our discussion to cells in a gradient of 0.5 nM/μm. **Figs 4C** and **5A** show that for this case, there is a clear difference in receptor occupancy in the front and back halves of the cell. However, for a shallow gradient, 0.1 nM/μm, there is significant overlap in the occupancy distributions for the two halves (**S2 Text, Section C**). Additionally, we have limited our analysis of the Ste2* distribution by dividing the cell into two halves. This division artificially introduces spatial information, because there are only two options for the direction of the gradient. In reality, the cell has no such spatial information; the full 2-dimensional distribution of active receptors must be considered to determine the direction of the gradient. As a measure of the cell’s estimate for the direction of the gradient, we use the direction of the vector that points from the origin (center of the cell) to the center of mass of the distribution of active receptors. We denote this vector as *g*_*est*_. In **Fig 6A and 6D**, we present a phase plane of the azimuthal angle and elevation (polar angle) of *g*_*est*_. The elevation measures the angle of *g*_*est*_ relative to the x-y plane. The azimuthal angle is the counterclockwise angle of *g*_*est*_ in the x-y plane away from the positive x-axis. *g*_*est*_ coincides with the true direction of the gradient ($\hat{x}$) at the point (0, 0). In **Fig 6A**, we plot a trajectory of *g*_*est*_ for a single cell. **Fig 6D** shows the time-averaged trajectories of all sixteen cells for an averaging window of 10 min. The remaining panels in **Fig 6**report the angle between *g*_*est*_ and the true direction of the gradient ($\hat{x}$), which we call “angular deviation”. An angular deviation of 0 indicates *g*_*est*_ is aligned with the gradient and a value of 180 indicates *g*_*est*_ is pointed in the $\text{-}\hat{x}$ direction. In **Fig 6B**, we plot the angular deviation for all 16 cells at each time point. We also plot the distribution of the angular deviation (**Fig 6C**). **Fig 6E** shows trajectories for the time-averaged angular deviations and the corresponding distribution is shown in **Fig 6F**. By comparing the distributions (**Fig 6C and 6F**), we find time-averaging improves the likelihood that the average position, or center of mass, of Ste2* correctly points towards the gradient.

**Fig 7:**
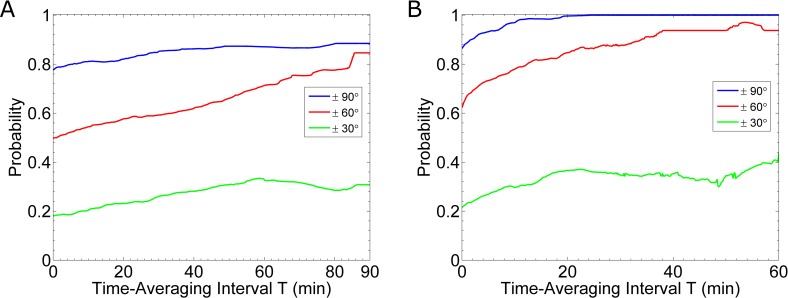
Time-averaging Gradient Prediction. Results for the probability that the cell’s prediction of the gradient is accurate within three thresholds: 90° (blue curves), 60° (red curves) and 30° (green curves). In all cases, we simulate cells in a 0.1 nM/μm gradient. **(A)** Results for slow binding kinetics. The curves are calculated from twenty-six simulations. (**B)** Results for fast binding kinetics. The curves are calculated from the same sixteen simulations used to generate **Fig 6**.

From the distributions, we can calculate the probability that the cell’s estimate of the gradient is accurate within a given threshold. For example, from the instantaneous distribution (**Fig 6C**), we find there is an 86% probability the cell’s estimate is within 90° of the gradient’s true direction. After time-averaging for 10 minutes (**Fig 6F**), this probability increases to 97%. **Fig 7**shows how this probability for selected thresholds improves with time-averaging. In the case of fast reaction rates: k_on_ = 1.6×10^6^ (M·s)^-1^ and k_off_ = 0.011 s^-1^, we find that after about 20 minutes of time-averaging, cells know the direction of the gradient within 90° (**Fig 7B**). In contrast, for the case of slow reaction rates: k_on_ = 1.6×10^5^ (M·s)^-1^ and k_off_ = 0.0011 s^-1^, if a cell time-averages for 90 minutes, then the probability of being correct within 90° is only 88% (**Fig 7A**). Similarly, we find short lengths of time-averaging (e.g. less than 20 min) are more beneficial in the case of fast reaction rates than slow rates. That is, for 20min of time-averaging, a system with fast rates improves the probability of being accurate within 60° by 22%; whereas, a system with slow rates improves the probability of being accurate within 60° by only 8%.

### Section III: Sensing during gradient formation

#### Formation of the gradient

**Fig 8:**
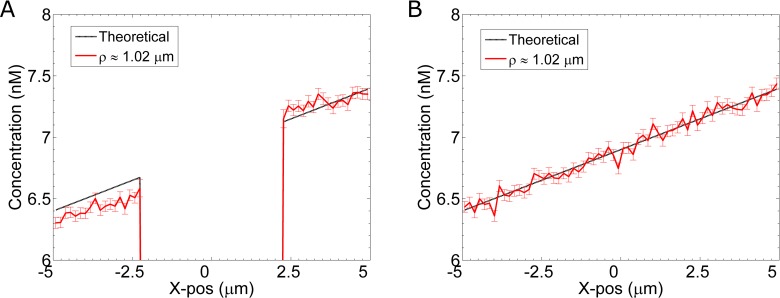
Steady-state Gradient Profiles when Molecules are only Injected from One Side. Pheromone molecules are added at the x = 5μm boundary and a partially absorbing boundary is placed at x = –5μm boundary (see Methods: “Gradient Method 2”). **(A)** The steady state concentration profiles in the presence of a cell. We do not simulate any reactions. **(B)** The steady state concentration profile in the absence of a cell. Note that the presence of the cell sharpens the gradient and reduces the overall concentration behind the cell.

We have thus far only considered steady state pheromone gradients. We now study the Ste2*distribution as the gradient forms across the cell. To simulate a developing gradient (0.1 nM/μm), we fix the pheromone concentration at the x = 5μm boundary and enforce a partially absorbing boundary at x = –5μm. That is, we do not inject new molecules from the x = –5μm boundary, but reflect molecules that reach this boundary back into the computational domain with the appropriate probability to establish the desired concentration at this boundary (see Methods: “Gradient Method 2” for details). **Fig 8B** shows the resulting steady state pheromone concentration profile in the absence of a cell. The inclusion of the cell boundary produces the pheromone concentration profile shown in **Fig 8A**. Again, we find the presence of the cell sharpens the gradient similar to previous cases (**Fig 4A and 4B**). However, unlike previous cases, the concentration is not held constant at x = –5μm, and the final concentration at this boundary depends on the presence or absence of the cell.

#### Transient differences in receptor occupancy

**Fig 9:**
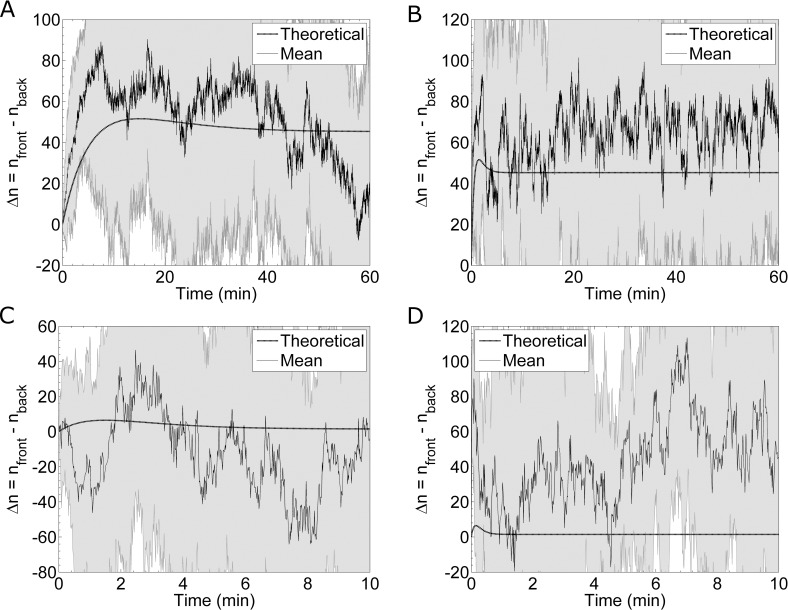
Transient Difference in Receptor Occupancy. Simulation results for the difference in receptor occupancy, Δn, between the front and back halves of a cell in a developing pheromone gradient of 0.1 nM/μm. The solid black lines are the mean Δn computed from simulations, and the shaded gray areas are the standard deviations from the means. The dashed black lines show the theoretical differences based on binding kinetics. **(A)** Results from twenty simulations of a system with slow binding kinetics in an average concentration of 6.9 nM. The theoretical curve is calculated using an average pheromone concentration of 6.775nM in the back half and 7.025nM in the front half. **(B)** Results from twenty simulations of a system with fast binding kinetics in an average concentration of 6.9nM. **(C)** Results from eight simulations of a system with slow binding kinetics in an average pheromone concentration of 69 nM. The theoretical curve is calculated using an average pheromone concentration of 68.875nM in the back half and 69.125nM in the front half. **(D)** Results from eight simulations of a system with fast binding kinetics in an average pheromone concentration of 69nM.

Recent work suggests that the largest difference in receptor occupancy (Δn = n_front_−n_back_) occurs transiently, before the receptor-pheromone system comes to equilibrium [13]. The receptor-pheromone binding and unbinding rates determine when this peak difference occurs (**Fig 9**) [13]. The authors conclude this transient effect is significant enough to improve the gradient sensing ability of yeast cells during mating. Furthermore, they argue this effect is particularly relevant for gradient sensing in high background levels of pheromone [13]. To investigate this transient effect, we simulate a cell whose receptors are all initially unoccupied and, using the method discussed above, flow pheromone from one side. We simulate cells in a shallow gradient of 0.1 nM/μm under two different background concentrations: 6.9nM (which is the K_D_ of the receptor) and 69nM (which is ten times the K_D_ of the receptor). Additionally, we study fast and slow binding kinetics. For each of these cases, **Fig 9**shows the theoretical and simulated Δn over time. Averaging over many simulations, we find the difference in occupancy is typically larger than theoretically expected (**Fig 9A, 9B and 9D**). The *in silico* Δn is higher than theoretically expected, because the simulations, which account for the physical boundary of the cell, produces a sharper gradient than the ideal gradient assumed in the theoretical model. Because our *in silico* model includes biophysical sources of noise, we can determine the fluctuations in Δn. These fluctuations appear much larger than the transient peak height in the theoretical model (**Fig 9**). Therefore, this transient effect is masked by the fluctuations in receptor occupancy.

**Fig 10:**
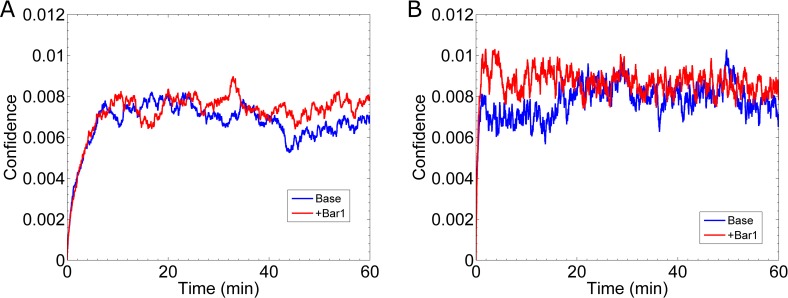
Gradient Estimation During Formation of the Gradient. **(A)** Result for the cell’s confidence (Eq 9) in the direction of the gradient for slow binding kinetics. In these simulations the emerging gradient is 0.1 nM/μm with a mean of 6.9 nM. The blue curve, labeled “Base”, is for the case without Bar1, and the red curve, labeled “+Bar1”, includes the effect of Bar1. The “Base” data is the mean of the same twenty simulations from Fig 9A, and the “+Bar1” data is the mean of sixteen simulations. **(B)** Same as **(A)** except for fast kinetics. The “Base” data is the mean of the same twenty simulations from Fig 9B, and the “+Bar1” data is the mean from sixteen simulations.

As discussed above, by analyzing the Ste2* distribution as two halves, we artificially introduce spatial information about the direction of the gradient in our analysis. A more appropriate measure of the Ste2*distribution that can be computed for a forming gradient is the center of mass of all Ste2* molecules. We interpret the direction of the resulting vector to be the cell’s estimate of the gradient’s direction. To construct a measure of the cell’s confidence in this estimate, we normalize the magnitude of this vector with respect to the fraction of active receptors:

$$\text{Confidence}=\frac{1}{R}\frac{n}{N}\left|\left\langle \vec{\text{Ste}2^{*}}\right\rangle \right|$$ (9)

where, *R* is the radius of the cell, *N* is the total number of receptors, and *n* is the number of active receptors. This confidence measure ranges between 0 and 1. A value near 0 indicates that either there are few active receptors or the active receptors are nearly uniformly distributed. A confidence value of 1 indicates all the receptors are active and located in the same position. For comparison, a cell at equilibrium in a uniform pheromone concentration of 6.9nM, the confidence is ≈ 6.4×10^−3^ (**S2 Text, Section D**). In the case of slow reaction rates, we find that the cell’s confidence reaches a maximum of 8 ×10^−3^ around 20min (**Fig 10A**). In the case of fast reaction rates, the maximum confidence is again around 8 ×10^−3^ and is reached slightly later around 30 min (**Fig 10B**). These results further demonstrate that transient effects do not significantly improve gradient sensing.

#### Bar1 improves gradient sensing for fast reaction rates

It has been shown that the pheromone protease Bar1 can improve the gradient sensing ability of the cell by locally sharpening the gradient [4,5]. We model the Bar1 concentration as a static field. That is, the Bar1 concentration is a function of the distance from center of the cell (see Methods: “Bar1 Model”). Based on previous models, we set this concentration of Bar1 at the cell surface to be 0.85nM [5]. Away from the cell surface, this concentration decreases as $\frac{1}{r}$. At each time step, pheromone molecules have a probability of being catalyzed based on the local concentration of Bar1. We use a catalytic rate of k_cat_ = 2.5×10^8^ (M·s)^-1^, which is based on a previous model [5].

In the case of slow reaction rates, the cell’s confidence (**Fig 10A**) is not significantly improved by the presence of Bar1. However, in the case of fast reaction rates, the cell’s confidence improves in the first 20 min to ≈ 8.9×10^−3^ with Bar1, as compared to 6.4×10^−3^ without Bar1 (**Fig 10B**). Thus, if the true binding and unbinding rates are slow, as measured [8,12], then Bar1 does not effectively help an isolated cell determine the direction of a shallow gradient.

## Discussion

### Fluctuations in receptor occupancy

One potential mechanism for detecting chemical gradients is for cells to use the spatial distribution of active receptors. However, fluctuations in binding and release of ligand and receptor diffusion introduce significant uncertainty in receptor occupancy, making this task more difficult. For a cell attempting to sense a shallow gradient, this uncertainty can mask the signal. For example, a cell with 10000 receptors in a shallow gradient of 0.1 nM/μm centered at the K_D_ of the receptor, has a difference in occupancy between the front and back of the cell of Δn ≈ 45 ± 50 [3]. This estimate does not include the effect of receptor diffusion, which further reduces the difference in receptor occupancy. Our results, which best match the theory of Berezhkovskii and Szabo [20], indicate that the effects from stochastic binding and unbinding are the largest source of variability in receptor occupancy.

### Mechanisms for noise-reduction

Because the cell receives all information on the extracellular pheromone concentration from the Ste2 receptor, fluctuations in receptor occupancy represent the first major source of noise during gradient sensing. Downstream signaling events could generate additional sources of noise and/or act to amplify gradients in receptor occupancy. However, these effects are beyond the scope of our current investigation. Instead, we evaluated potential cellular mechanisms cells might employ to reduce noise from fluctuations in receptor occupancy.

Recent work derives the relationship between the length of time-averaging and the amount of noise-reduction [20]. Simulations of our particle-based stochastic reaction-diffusion model corroborate these predictions (**Fig 3**). For example, using the measured rates, k_on_ = 1.6×10^5^ (M·s)^-1^ and k_off_ = 0.0011 s^-1^ [8,12], we find a yeast cell must wait 40min or longer to appreciably reduce fluctuations in receptor occupancy. Since yeast cells typically polarize and initiate growth within 20 – 30min of exposure to pheromone, it is unlikely a cell can sense a gradient as shallow as 0.1 nM/μm. Consistent with this observation, it has recently been shown that during chemotropic growth the polarity site is mobile and reorients toward the gradient when initial polarization is in the wrong direction [26,27]. Recent estimates of the reaction rates are an order of magnitude smaller than the rates above [13], which further reduces the likelihood that a yeast cell can sense shallow gradients before polarizing their growth.

Receptor endocytosis has also been suggested as a noise-reduction mechanism, because it removes ligand from the environment thereby eliminating noise from re-binding the same ligand molecule [6]. It may also serve to reduce noise due to receptor diffusion. To investigate the effects of endocytosis on gradient sensing, we adapted our model to absorb rather than release pheromone molecules. Contrary to expectations, we do not find any advantage to removing the ligand. That is, ligand absorption does not reduce the noise as compared to releasing ligand **(Fig 3C and 3D**).

The pheromone protease, Bar1, has also been suggested as a possible mechanism to improve gradient sensing. We implicitly modeled Bar1 as a concentration field, which radially decays away from the cell’s surface. Our results indicate that Bar1 does improve the cell’s ability to sense an emerging gradient (**Fig 10B**). Specifically, during the first 20 min that a shallow gradient (0.1 nM/μm) forms around the cell, the presence of Bar1 increases the cell’s confidence in estimating the direction of the gradient. Curiously, our results predict this advantage only occurs if the reaction rates for Ste2 and pheromone are fast (k_on_ = 1.6×10^6^ (M·s)^-1^ and k_off_ = 0.011 s^-1^). This advantage may also be dependent upon other parameters, e.g. the concentration of Bar1 near the cell and the catalytic rate of Bar1 on pheromone. Future work will be dedicated to studying how these parameters affect gradient sensing.

### Gradient sharpening due to steric effects

Because we directly simulate diffusion, our method captures subtle effects in the distribution of pheromone, e.g. boundary effects from the cell membrane. Similar effects have been shown to play a significant role in other systems, for example in the arrangement of actin bundles in filopodia [28]. Here, we found that because the cell is impermeable to pheromone, it acts to sharpen the gradient (**Figs 4A and 4B**and **8**). Importantly, we find this sharpening is reflected in the active receptor distribution (**Figs 4C and 4D**and **9**). Our simulation methods are analogous to some yeast gradient sensing experiments, in which a pheromone gradient is established by flowing two different concentrations of pheromone into a microfluidics chamber [5,26,29–31]. In those experiments, it is not uncommon for multiple cells to be adjacent, e.g. as mother-daughter pairs or as multi-cell clusters. Our results suggest these adjacent cells experience a sharper than expected gradient. Additionally, we performed simulations using small (1.75 μm radius) cells and found less gradient sharpening than with 2.5 μm radius cells. Consequently, these cells had less of a difference in receptor occupancy (**S2 Text, Section E**). Our results complement other work which corroborates this relationship between cell size and the cell’s ability to gradient sense [32].

Gradient sharpening is strongest if pheromone is injected into the computational domain from only one side. This arrangement is common in microfluidic gradient chambers [30,33]. Additional steric effects may also be present due to the microfluidic chambers themselves. For example, many microfluidic chambers have a height similar to a yeast cell (~5μm). Therefore, the presence of a cell severely impedes the flow of pheromone in the chamber and further sharpens the gradient. Hence experiments that aim to test the limit of gradient sensing (i.e. the shallowest gradient a cell can detect) must carefully consider steric effects from the cell itself, any adjacent neighbors and the experimental tools. These effects alter the gradient, such that the true gradient experienced by the cells is sharper than expected.

### Tools for simulating gradients

While we are motivated by understanding the mechanisms used by yeast to detect gradients of pheromone, our simulation methods should find applications in other contexts. We derive two methods for generating a linear particle gradient in 3-dimensional particle-based stochastic diffusion simulations. These methods explain how many and how far to inject particles into the simulation space per time step. We also capitalize on hardware acceleration via the use of GPGPUs to achieve massive parallelization. As a result we were able to simulate tens of thousands of molecules over billions of time steps.

## Methods

Our goal is to develop a simulation platform that resolves individual signaling molecules in a continuous 3-dimensional space (**Fig 1**). It also should faithfully capture the stochastic properties of diffusion of both the extracellular signaling molecules and receptors in the cell membrane, and of the biochemical reactions involved in ligand binding and release and receptor internalization (**Fig 1A**). For these reasons, we choose a Particle-Based Stochastic Reaction-Diffusion model in which molecules are modeled as point particles that can stochastically react and diffuse continuously in space.

Our simulation domain is a cubic volume with 10μm sides. We model the cell as a sphere at the center of the volume with a radius of 2.5μm (**Fig 1B**). The state of the system is defined by the position of all the molecules and chemical state of each receptor (bound or unbound). Pheromone molecules cannot be located inside the cell, and receptor molecules are restricted to the surface of the cell. Given the current state at time t_0_, we determine the subsequent state at time t_0_ + Δt, where Δt is a time step of fixed length, by calculating all binding reactions, unbinding reactions and diffusion of each molecule. A binding event occurs with probability P_bind_ (**Table 1**), if a pheromone molecule is within a distance of r_bind_ = 4nm (binding radius) of an unbound receptor (**Fig 1B.W**). An unbinding event occurs during the time step, Δt, with probability P_unbind_ (**Table 1**). A bound receptor releases a pheromone molecule a distance of r_unbind_ = 4nm (unbinding radius) away at a randomly chosen angle (**Fig 1B.W**). During diffusion, each particle stochastically moves to a new position with appropriate conditions enforced at the boundaries of the computational domain and the cell (**Fig 1B.X‒1B.Z**). The time step is chosen such that the length scale of pheromone diffusion ($\sqrt{2D_{\alpha }\Delta t}$) is similar to the binding radius (4nm). We have performed extensive tests to validate all our simulation methods, including tests to demonstrate that: 1) diffusion produces the correct mean squared displacement; 2) first order reactions match theoretical decay curves, and 3) second order reactions match theoretical binding curves. We do not include these tests here, because each process has been extensively validated both theoretically and computationally by others [18], and we did not find our validations particularly insightful. On the microscopic scale, our model captures the stochasticity due to reactions and diffusion and monitors the exact position of all molecules in a 3D space. Below, we explain the microscopic rules that each molecule follows.

### Binding reactions

In most particle-based reaction-diffusion simulations, a binding event is executed as follows. When a ligand molecule is nearby (within the binding radius of) an unbound receptor molecule, the ligand molecule is removed from the system, and the receptor molecule is switched to the ‘bound’ state. To match the macroscopic binding kinetics, the binding radius is calculated from the binding rate and the diffusion constants of the two molecular species:

$$r_{\text{bind}}=\frac{k_{\text{on}}}{4\pi \left(D_{\alpha }+D_{\text{Ste}2}\right)}$$ (10)

This expression can be derived from Fick’s Law of diffusion and works well when the reaction is diffusion limited. Because α-factor binding to Ste2 is not diffusion limited, to match the rates reported in the literature using this method requires a binding radius on the order of Angstroms, which is much smaller than the size of Ste2 (GPCRs protrude about 4nm outside the cell membrane [35]). As discussed by Erban and Chapman the unrealistic binding radius results from the assumption that the binding probability is 100% [18]. That is, a ligand molecule within the binding radius of an unbound receptor binds with certainty. The model put forward by Erban and Chapman, removes this assumption and establishes a mathematical framework, in which the binding probability is a function of the binding radius [18,36]. That is, a ligand molecule within a specified binding radius binds with a probability that produces an average binding rate consistent with the macroscopic rate constant k_on_. We choose the binding radius to be 4nm and calculate the binding probability by numerically solving the following system of equations derived by Erban and Chapman [36]:

$$\frac{k_{\text{on}}\Delta t}{r_{\text{bind}}{}^{3}}=P_{\text{bind}}\int_{0}^{1}4\pi z^{2}g\left(z\right)dz$$ (11)

where,

$$g(\hat{r})=(1-P_{bind})\int_{0}^{1}K(\hat{r},{\hat{r}}^{\prime },\gamma )g({\hat{r}}^{\prime })d{\hat{r}}^{\prime }+\int_{1}^{\infty }K(\hat{r},{\hat{r}}^{\prime },\gamma )g({\hat{r}}^{\prime })d{\hat{r}}^{\prime }+\frac{P_{bind}K(\hat{r},\alpha ,\gamma )}{\alpha^{2}}\int_{0}^{1}g(z)z^{2}dz$$

$$K(z,z^{\prime },\gamma )=\frac{z^{\prime }}{z\gamma \sqrt{2\pi }}\left(\exp\left[-\frac{{\left(z-z^{\prime }\right)}^{2}}{2\gamma^{2}}\right]-\exp\left[-\frac{{\left(z+z^{\prime }\right)}^{2}}{2\gamma^{2}}\right]\right)$$

$$\alpha =\frac{r_{unbind}}{r_{bind}}$$

$$\gamma =\frac{\sqrt{2(D_{\alpha }+D_{Ste2})\Delta t}}{r_{bind}}$$

Using the values for Δt, k_on_, D_α_, D_Ste2_, r_bind_ and r_unbind_ given in **Table 1**, a pheromone molecule has a 0.2% chance of binding. We provide a detailed description of how we calculate the probability in the Supplemental Methods (**S1 Text, Section A**).

Importantly, we note that to apply the method of Erban and Chapman to our system, we must double the binding probability. The probability calculated from their method is appropriate when the ligand molecule can approach the receptor from any direction. However, in our system, the pheromone molecules can only approach the receptor from the outside of the cell. Therefore to achieve macroscopic rate constants consistent with experimental measurements, we double the binding probability. This adjustment was also used in recent work [37]. See the Supplemental Methods (**S1 Text, Section A**).

### Unbinding reactions

Given a dissociation rate k_off_, we can calculate the probability, P_unbind_, that a ligand molecule dissociates from a ‘bound’ receptor in the time interval Δt as follows:

$$P_{\text{unbind}}=1-\exp\left[-k_{\text{off}}\;\Delta t\right]$$ (12)

A new pheromone molecule is created a fixed distance, r_unbind_, and in a random direction from the receptor (**Fig 1B.W**). As with the binding radius, we take r_unbind_ = 4nm. We do not allow a pheromone molecule to be released inside the cell. Lastly, the receptor molecule is switched to the ‘unbound’ state.

### Diffusion of pheromone

Let (x(t), y(t), z(t)) be the current position at time t, then to diffuse a pheromone molecule in 3D, the new position (x(t + Δt), y(t + Δt), z(t + Δt)) is found from the following equations:

$$x(t+\Delta t)=x(t)+Z_{1}\sqrt{2D\Delta t}$$ (13a)

$$y(t+\Delta t)=y(t)+Z_{2}\sqrt{2D\Delta t}$$ (13b)

$$z(t+\Delta t)=z(t)+Z_{3}\sqrt{2D\Delta t}$$ (13c)

The Z_i_s are independent random numbers drawn from a Gaussian distribution with a mean of 0 and a variance of 1. The new position is modified if it is located outside the simulation volume or inside the cell. Reflecting boundary conditions are imposed at the four boundaries: *y* = ±5μm and *z* = ±5μm (**Fig 1B.X**). Additionally, pheromone molecules reflect off the surface of the cell, because the cell membrane is impermeable to pheromone (**Fig 1B.Y**). Details for calculating the reflection off the cell surface are provided in the Supplemental Methods (**S1 Text, Section B**).

The last two boundaries, *x* = ±5μm, are constructed to establish a linear pheromone gradient along the x-axis. We describe two different methods for treating the *x* = ±5μm boundaries. In method 1, each boundary has a fixed concentration. In method 2, one boundary has a fixed concentration while the other is partially absorbing. The next two sections describe the physical interpretation and algorithmic implementation for each method.

### Pheromone gradient method 1 − fixed concentrations

In this method, we model a fixed concentration at each boundary. A gradient is formed when we set the concentration at one end of the computational domain higher than at the other. This method is consistent with the design of many microfluidic chambers used to study gradient sensing [5,26,29–31]. To maintain a fixed concentration at each boundary, pheromone molecules are added to and removed from the simulation volume in processes called ‘injection’ and ‘ejection’, respectfully (**Fig 1B.Z**).

For ejection, we remove all pheromone molecules located outside the boundaries (*x* < –5μm, or *x* > 5μm) (**Fig 1B.Z**). For injection, we create a number of new pheromone molecules and position them near either the *x* = 5μm or *x* = –5μm boundary. On average, for a concentration *c* at the boundary, the number to inject at each time step is calculated using the equation:

$$n_{\text{inj}}=\frac{0.6022}{nM∙\mu m^{3}}∙c∙a\sqrt{\frac{D_{\alpha }\Delta \tau }{\pi }}$$ (14)

where *a* is the area of the boundary (100μm^2^ for most of our simulations); D_α_ is the diffusion constant for pheromone molecules, and Δτ is the elapsed time between two injection processes. The derivation of Eq 14 is found in the Supplemental Methods (**S1 Text, Section C**). Although Eq 14 provides the average number of molecules to be injected, due to the stochastic nature of diffusion, the actual number injected can vary for a given time step. During an injection step, the number to inject is a random number drawn from a Poisson distribution with a mean of n_inj_. The position of a newly injected molecule is also determined randomly. The y and z positions are determined from a uniform probability distribution across their respective domains (e.g between –5μm and 5μm, inclusively). The x position is calculated as a random distance, called the ‘injection distance’, into the simulation volume from the boundary (**Fig 1B.Z**). The probability distribution function for the injection distance, d_inj_, is given by:

$$P\left(d_{\text{inj}}\right)=\frac{1}{2}\left[1-\text{erf}\left(\frac{d_{\text{inj}}}{\sqrt{4D_{\alpha }\Delta \tau }}\right)\right]$$ (15)

The derivation of Eq 15 is provided in the Supplemental Material (**S1 Text, Section C**). Because it is computationally expensive to generate a random number from the distribution given by Eq 15, we select a random value from a pre-calculated list. This list has more than 12 million random values whose distribution matches Eq 15. Further discussion for implementing Eq 15 is provided in the Supplemental Methods (**S1 Text, Section C**).

For computational efficiency, these two processes (injection and ejection) are implemented on a slightly coarser time scale, Δτ, than the time scale for diffusion Δt. It is important to note that ejection and injection must be calculated on the same time scale. Details and justification for the two time scales are discussed below in “Algorithm Overview”.

### Pheromone gradient method 2 –partially absorbing boundary

In this method, we model a fixed concentration at one boundary (*x* = 5μm), while the other boundary (*x* = –5μm) is partially absorbing. We use this method to simulate the formation of a gradient from a source located at the positive x boundary (see Results Section III).

At the *x* = 5μm boundary, the average number of molecules to inject at each time step, n_inj_, is given by:

$$n_{\text{inj}}=\frac{0.6022}{nM∙\mu m^{3}}∙\left(c_{x=5}∙a\sqrt{\frac{D_{\alpha }\Delta \tau }{\pi }}+\frac{1}{2}a∙g∙D_{\alpha }\Delta \tau \right)$$ (16)

The derivation of Eq 16 is found in the Supplemental Methods (**S1 Text, Section C**). Note that in addition to defining the desired concentration at the boundary, *c*_*x = 5*_, we also define the desired gradient at the boundary: *g*. Eq 14 is a special case of Eq 16, in which there is no gradient (*g* = 0 nM/μm) outside our volume (*x* > 5μm).

At the *x* = –5μm boundary, no new molecules are injected, and during ejection, not all molecules located outside the boundary (*x* < –5μm) are removed. Instead, each pheromone molecule has a probability of being reflected back inside the volume; otherwise, the molecule is removed. To achieve a steady state gradient of *g*, the probability of reflection is given by:

$$P_{\text{Ref}}=1-\frac{g}{c_{x=-5}\sqrt{\frac{1}{\pi D_{\alpha }\Delta \tau }}+\frac{1}{2}g}$$ (17)

if and only if,

$$\frac{c_{x=-5}}{g}\gg \sqrt{4D_{\alpha }\Delta \tau }$$

The derivation of Eq 17 is found in the Supplemental Methods (**S1 Text, Section C**). The concentration, *c*_*x* = –5_, and gradient, *g*, are the steady state concentration and gradient at the *x* = –5μm boundary when no cell is present in the computational domain. In the presence of a cell the pheromone molecules coming from the opposite boundary must diffuse around the cell. Therefore in this situation the resulting concentration will be less than *c*_*x* = –5_, and the gradient will be steeper than *g*.

As in method 1, the injection and ejection processes are implemented on a slightly coarser time scale, Δτ, than the primary time scale: Δt. Because these processes are calculated less frequently, our program is more computationally efficient.

### Diffusion of receptors

Each receptor molecule, ‘bound’ or ‘unbound’, diffuses on the cell membrane (**Fig 1B.Y**). We approximate diffusion on this surface by first diffusing the receptor in 3-dimensions and then projecting the receptor back onto the surface of the cell. This approach is computationally efficient and accurate for small time steps. The details of these two steps for diffusing a receptor molecule are as follows. First, a new position is calculated using Eq 13 and a diffusion constant appropriate for proteins in the plasma membrane (D = 0.0025 μm^2^/s) [34]. Let this new position be $\left(\tilde{x},\tilde{y},\tilde{z}\right)$. Second, we project $\left(\tilde{x},\tilde{y},\tilde{z}\right)$ onto the surface of the cell, which is modeled as a sphere of radius *R* and centered at the origin (0, 0, 0) using the equations:

$$\tilde{r}=\sqrt{{\tilde{x}}^{2}+{\tilde{y}}^{2}+{\tilde{z}}^{2}}$$ (18a)

$$x(t+\Delta \tau )=\tilde{x}\frac{R}{\tilde{r}}$$ (18b)

$$y(t+\Delta \tau )=\tilde{y}\frac{R}{\tilde{r}}$$ (18c)

$$z(t+\Delta \tau )=\tilde{z}\frac{R}{\tilde{r}}$$ (18d)

The derivation of Eq 18 is provided in the Supplemental Methods (**S1 Text, Section D**). For computational efficiency, we diffuse the receptors on a slightly coarser time scale, Δτ, than the primary time scale: Δt. We do not sacrifice much accuracy, because the diffusion constant for membrane-bound receptors is small compared to that of extracellular pheromone molecules.

### Algorithm overview

Our simulation algorithm and order of operations closely follows the general algorithm described by [36]. We modify their algorithm, because of the spatial domains (outside or on the cell) and non-uniform distribution of molecules (the ligands have a linear concentration gradient and the receptors are restricted to the surface of the sphere). Our simulation algorithm is broken into the six processes described above: binding reactions, unbinding reactions, diffusion of pheromone molecules, ejection of pheromone molecules, injection of pheromone molecules and diffusion of receptors. The last three processes are simulated on a coarser time scale (time step = Δτ) than the first three processes (time step = Δt). Below, we provide pseudo-code of our simulation algorithm.

**At each time** **step**

**I.    Binding Reactions**–Each ‘unbound’ receptor has a chance to bind a single, nearby pheromone molecules.

**II.    Unbinding Reactions**–Each ‘bound’ receptor, including those from step **1)**, has a chance to release its pheromone molecule.

**III.    Pheromone Diffusion**–Each pheromone molecule diffuses. They reflect off the cell’s surface, and the y = ±5μm and z = ±5μm boundaries.

**After every** **(Δτ/Δt) steps**

**IV.    Receptor Diffusion**–Each receptor molecule diffuses on the surface of the cell.

**V.    Ejection**–Remove all pheromone molecules outside the x = ±5μm boundaries.

**VI.    Injection**–Add new pheromone molecules near the x = ±5μm boundaries.

We choose the coarse time scale, Δτ, based on two criteria. One, receptor diffusion should not be many times larger than the binding radius. And two, the average injection distance should be much less than 2.5μm, which is the distance from the *x* = ±5μm boundaries to the cell. For Δτ = 50μs, receptors diffuse about 0.5nm, which is much smaller than 4nm. Also for Δτ = 50μs, the average injection distance is about 0.05μm, which is much smaller than 2.5μm.

While our model is derived from physical processes, it can be thought of as an Agent-Based Model; wherein, we simulate 10,000–20,000 molecules (the “agents”), each of which follows a set of rules. Because many of these rules are independent of other molecules, we can parallelize the algorithm at each process. For example, during pheromone diffusion, process **III**, a new position is calculated for each pheromone molecule. This calculation is independent from all other molecules. Hence, the diffusion of many pheromone molecules can be calculated simultaneously. Ideally, we would calculate the new position of every pheromone molecule in parallel. To achieve massive parallelization with minimal coding effort, we turn to Hardware Acceleration using NVIDIA GPUs. We write the program in CUDA C, which is an extension of the C Programming Language, developed by NVIDIA to facilitate High Performance Computing on their GPGPUs. Each of the six processes is executed on the GPU, one at a time, as arranged above, in order to ensure all reactions are complete before the molecules diffuse. That is to say, there is a global synchronization between processes. All simulations were run on UNC’s KillDevil cluster, which has 2.67GHz Intel processors connected to NVIDIA M2070 GPUs. In a typical simulation, we simulate roughly 17,000 particles for 3.6 billion time steps (1 hour at 1μs time steps), which takes about 34 hours to complete. The parallelization offers scalability in complexity but not a corresponding increase in computation time. For example, a simulation similar to the one above, but with double the ligand concentration, takes about 45 hours: a 32% increase. Using the CUDA nomenclature, the binding, unbinding and receptor diffusion processes launch 53 Blocks per Grid and 192 Threads per Block. The pheromone diffusion and ejection processes launch 18 Blocks per Grid and 256 Threads per Block; the injection process launches 2 Blocks per Grid (one each for x = ±5μm boundary) and 256 Threads per Block.

### Receptor cycling model

To determine if receptor cycling can reduce noise, we developed a simplified model of receptor cycling. We compare simulations of the basic model described above, in which receptors bind and unbind pheromone, to simulations of the simplified receptor cycling model, in which receptors bind pheromone and are endocytosed. Specifically, an active (pheromone-bound) receptor, Ste2*, can be endocytosed and replaced with an unbound receptor, Ste2. The new Ste2 molecule is added to a random position on the cell surface. This method of endocytosis with immediate replacement keeps the total number of receptors constant, and allows us to directly compare results from this model to results from the basic model. Although the endocytosis rate for Ste2* is 0.0021 s^-1^ [14,15], we use a rate of 0.0011 s^-1^, which is the unbinding reaction rate from the basic model (See Table 1). In our algorithm, process **II** (Unbinding Reactions) is replaced with the following.

**II. Endocytosis Reactions**–Each ‘bound’ receptor, including those from step **1)**, has a chance to be endocytosed.

### Bar1 model

To determine if the pheromone protease Bar1 can reduce noise and improve gradient sensing, we have also developed a simplified model for a cell releasing Bar1. In addition to the basic model described above, in which receptors bind and unbind pheromone, we include the reaction of Bar1 degrading pheromone. In principle this catalytic reaction can be modeled much like the binding reaction; that is, individual Bar1 molecules could be simulated and have a probability of degrading nearby pheromone molecules. However we choose to avoid the computational cost of this method. Instead, we model Bar1 concentration as a static, radial field extending from the surface of the cell:

$$\left[\text{Bar}1\right]\left(r\right)={\left[\text{Bar}1\right]}_{0}\frac{R}{r}$$ (19)

where *r* is the distance from the center of the cell; *R* is the radius of the cell, and [Bar1]_0_ is the Bar1 concentration at the surface of the cell. Similar to previous modeling work, we set [Bar1]_0_ = 0.85nM [5]. Based on their distance from the cell surface, pheromone molecules have a probability of being degraded as given by:

$$P_{\text{cat}}\left(r\right)\approx k_{\text{cat}}∙\left[\text{Bar}1\right]\left(r\right)∙\Delta t$$ (20)

As modeled previously, we set the catalytic reaction rate, k_cat_, to be 2.5e8 (M·s)^-1^ [5]. In our algorithm, the process for Bar1-mediated catalytic reaction is inserted between process **II** (Unbinding Reactions) and **III** (Pheromone Diffusion). Here, we label the Bar1 process as **IIB**.

**IIB. Catalytic Reactions**–Each pheromone molecule has a chance, based on its current position, of being degraded.

## Supporting information

S1 Text Supplemental Methods. This text describes how we calculate the binding probability (Section A), calculate pheromone molecules reflecting off the cell surface (Section B), derive and calculate the injection rate, injection distance and reflection probability (Section C), calculate receptor diffusion on the cell surface (Section D) and calculate pheromone gradient profiles (Section E). Each of these sections is referenced in the manuscript where appropriate.
(DOCX)

S2 Text Supplemental Results. This text contains some additional supporting results, including gradient sharpening in a larger simulation volume (Section A), gradient sharpening in simulations with “slow” reaction rates (Section B), receptor occupancies in the front and back halves of the cell in a shallow gradient (Section C), a negative control for our confidence measure (Section D) and results from simulations with smaller cells of radius 1.75μm (Section E).
(PDF)

S1 Movie Example of Receptor Diffusion. This movie shows receptors diffusing on the surface of the cell, as simulated with our model. Neither pheromone molecules nor reactions were simulated to generate the data for this movie. Positions of active receptors are indicated by green points, and positions of inactive receptors are indicated by red points. The movie is constructed in Matlab; the surface of the cell is shown as a grey sphere.
(MPG)

S2 Movie Example of Pheromone Diffusion and Gradient Method 1. This movie visualizes a short simulation from three viewpoints of pheromone molecules diffusing in the computational volume. The positions of pheromone molecules are shown as blue points, and the impermeable cell surface is shown as a grey sphere. No receptors, and therefore no reactions, were simulated in this example. Initially, the simulation begins with no pheromone molecules; new molecules are added as prescribed by Gradient Method 1. That is, new molecules are added at the *x* = –5 μm and *x* = 5μm boundaries. This movie shows a simulation of a 1.0 nM/μm gradient (steeper than any gradient simulated for the data in the main text). The first 7 seconds of the video show the first 5ms of simulation time; afterwards, the video jumps to the 0.5s mark of the simulation (see simulation time indicator in the video).
(MP4)

S3 Movie Example of Pheromone Diffusion and Gradient Method 2. This movie is similar to Movie S2, with the exception that it shows pheromone molecules being injected as prescribed by Gradient Method 2. That is, new molecules are added at the *x* = 5μm boundary, and pheromone molecules outside the *x* = –5μm boundary are partially reflected.
(MP4)

S1 DataSet Figs 2 & 3. This ZIP archive contains Matlab formatted data files and Matlab scripts (with instructions) needed to generate the curves shown in Figs 2 & 3. This archive also contains a DOCX file with more detailed information. See also ‘S5 DataSet’, ‘S6 DataSet’ and ‘S7 DataSet’.
(ZIP)

S2 DataSet Figs 4, 5 & 8. This ZIP archive contains Matlab formatted data files and Matlab scripts (with instructions) needed to generate the curves shown in Figs 4, 5 & 8. This archive also contains a DOCX file with more detailed information. See also ‘S8 DataSet’, ‘S9 DataSet’, ‘S10 DataSet’, ‘S11 DataSet’ and ‘S12 DataSet’.
(ZIP)

S3 DataSet Figs 6, 7 & 10. This ZIP archive contains Matlab formatted data files and Matlab scripts (with instructions) needed to generate the curves shown in Figs 6, 7 & 10. This archive also contains a DOCX file with more detailed information.
(ZIP)

S4 DataSet Fig 9. This ZIP archive contains Matlab formatted data files and Matlab scripts (with instructions) needed to generate the curves shown in Fig 9. This archive also contains a DOCX file with more detailed information.
(ZIP)

S5 DataSet Additional Fig 2. This ZIP archive contains a Matlab formatted data file needed to generate Fig 2; this data should be used in conjunction with the files in ‘S1 DataSet’.
(ZIP)

S6 DataSet Data for Fig 3C part 1. This ZIP archive contains a Matlab formatted data file needed to generate the blue curve in Fig 3C. This data should be combined with the file from ‘S7 DataSet’ before being used with the files in ‘S1 DataSet’; the script needed to combine the two files is included here.
(ZIP)

S7 DataSet Data for Fig 3C part 2. This ZIP archive contains the Matlab formatted data file to be combined with the file in ‘S6 DataSet’.
(ZIP)

S8 DataSet Data for Fig 4A. This ZIP archive contains a Matlab formatted data file needed to generate Fig 4A; this data should be used in conjunction with the files in ‘S2 DataSet’.
(ZIP)

S9 DataSet Data for Fig 8A part 1. This ZIP archive contains a Matlab formatted data file needed to generate Fig 8A. This data should be combined with the file from ‘S10 DataSet’ before being used with the file in ‘S2 DataSet’; the script needed to combine the two files is included here.
(ZIP)

S10 DataSet Data for Fig 8A part 2. This ZIP archive contains the Matlab formatted data file to be combined with the file in ‘S9 DataSet’.
(ZIP)

S11 DataSet Data for Fig 8B part 1. This ZIP archive contains a Matlab formatted data file needed to generate Fig 8B. This data should be combined with the file from ‘S11 DataSet’ before being used with the file in ‘S2 DataSet’; the script needed to combine the two files is included here.
(ZIP)

S12 DataSet Data for Fig 8B part 2. This ZIP archive contains the Matlab formatted data file to be combined with the file in ‘S10 DataSet’.
(ZIP)
