# Supplementary material for: Testing the limits of gradient sensing
Source: PLoS Comput Biol. 2017 Feb 16;13(2):e1005386. doi: 10.1371/journal.pcbi.1005386 (PMC5347372; doi:10.1371/journal.pcbi.1005386)
Supplement: S1 Text — This text describes how we calculate the binding probability (Section A), calculate pheromone molecules reflecting off the cell surface (Section B), derive and calculate the injection rate, injection distance and reflection probability (Section C), calculate receptor diffusion on the cell surface (Section D) and calculate pheromone gradient profiles (Section E). Each of these sections is referenced in the manuscript where appropriate. (DOCX) [file pcbi.1005386.s001.docx]

“Testing the Theoretical Limits of Gradient Sensing in Yeast”

V Lakhani and T Elston

Text S1: Supplemental Methods

1. Calculating the Binding Probability ……………………………………………………………… 2
2. Calculating Pheromone Reflection off the Cell Surface …………………………………………. 7
3. Injection Boundary Condition: Derivation of Eqs 5 – 8 ………………………………………… 9
   1. Gradient Method 1 ……………………………………………………………………… 9
   2. Injection Distance ……………………………………………………………………... 11
   3. Gradient Method 2 ……………………………………………………………………. 13
4. Receptor Diffusion on the Cell Surface: Derivation of Eqn 9 ………………………………….. 15
5. Measuring the Pheromone Distribution ………………………………………………………… 16

***A. Calculating the Binding Probability***

To simulate the second-order reaction of a pheromone molecule binding to a Ste2 receptor, we implement the $\lambda-\bar{\varrho}$ method developed by Erban and Chapman [1]. The equations presented here, and the technique used to numerically solve them, are taken directly from their paper. We deviate slightly from their exact technique, in that we 1) choose a different numerical integration method and 2) double the binding probability calculated from their method (**Fig A1**).

We use the biophysical parameters defined in **Table 1**. In particular, we use the binding rate (k_on_), unbinding rate (k_off_) and diffusion constants for pheromone (D_α_) and the receptor (D_Ste2_). Furthermore, we choose a time step (Δt), binding radius (r_bind_) and unbinding radius (r_unbind_). The binding and unbinding parameters are chosen based on the size of the receptor. The time step is chosen such that the combined diffusion step size is not much greater than the binding radius. That is, the dimensionless parameter $\gamma$ (Eqn A1) is not much greater than $1$. In our simulations, $\gamma\approx4$.

|  | $\gamma= \frac{\sqrt{2\left( D_{\alpha}+D_{Ste2} \right)\Delta t}}{r_{\mathrm{bind}}}$ | Eqn A1 |
| --- | --- | --- |

Ideally, Δt would be much smaller, so that, $\gamma\ll1$. However, this time step is too small for practical computation, especially because we aim to simulate the system for one hour. Hence, because $\gamma>1$, our chosen time step is considered “large”. Nonetheless, according to the $\lambda-\bar{\varrho}$ method, we can calculate the binding probability (P_bind_) based on the parameters listed above.

The dimensionless reaction rate, κ (Eqn A2), is related to the binding probability by Eqn A3, which describes the rate of removing particles in one time step.

|  | $\kappa= \frac{k_{\mathrm{on}}\Delta t}{{r_{\mathrm{bind}}}^{3}}$ | Eqn A2 |
| --- | --- | --- |
|  | $\kappa- P_{\mathrm{bind}}\int_{0}^{1} 4\pi z^{2}g\left( z \right)dz = 0$ | Eqn A3 |

The function $g\left( \hat{r} \right)$ is defined below (Eqn A4).

|  | $g\left( \hat{r} \right)=\left( 1-P_{\mathrm{bind}} \right)\int_{0}^{1} K\left( \hat{r},\hat{r}^{'},\gamma\right)g\left( \hat{r}^{'} \right)d\hat{r}^{'}$  $+ \int_{1}^{\infty} K\left( \hat{r},\hat{r}^{'},\gamma\right)g\left( \hat{r}^{'} \right)d\hat{r}^{'}+ \frac{P_{\mathrm{bind}}K\left( \hat{r},\alpha,\gamma\right)}{\alpha^{2}}\int_{0}^{1} g\left( z \right)z^{2}dz$ | Eqn A4 |
| --- | --- | --- |

The parameter α is the dimensionless ratio of the binding and unbinding radii (Eqn A5). The Kernel $K\left( \hat{r},\hat{r}^{'} \right)$ is derived from Green’s function for diffusion (Eqn A6).

|  | $\alpha= \frac{r_{\mathrm{unbind}}}{r_{\mathrm{bind}}}$ | Eqn A5 |
| --- | --- | --- |
|  | $K\left( z,z^{'},\gamma\right)=\frac{z^{'}}{z\gamma\sqrt{2\pi}}\left( \exp\left[ -\frac{\left( z-z^{'} \right)^{2}}{2\gamma^{2}} \right]-\exp\left[ -\frac{\left( z+z^{'} \right)^{2}}{2\gamma^{2}} \right] \right)$ | Eqn A6 |

To determine P_bind_ numerically, we use the Secant Root-Finding Method on Eqn A3, which also requires numerically evaluating Eqn A4. We truncate the semi-infinite integral (second term in Eqn A4) and approximate $g\left( \hat{r} \right)=1$ for $\hat{r}\geq S$ (Eqn A7). We choose $S=5\gamma\approx20$.

|  | $g\left( \hat{r} \right)=\left( 1-P_{\lambda} \right)\int_{0}^{1} K\left( \hat{r},\hat{r}^{'} \right)g\left( \hat{r}' \right)d\hat{r}' + \int_{1}^{S} K\left( \hat{r},\hat{r}^{'} \right)g\left( \hat{r}' \right)d\hat{r}'+ \int_{S}^{\infty} K\left( \hat{r},\hat{r}^{'} \right)d\hat{r}' + \frac{P_{\lambda}K\left( \hat{r},\alpha\right)}{\alpha^{2}}\int_{0}^{1} g\left( \hat{r}^{'} \right){\hat{r}^{'}}^{2}d\hat{r}^{'}$ | Eqn A7 |
| --- | --- | --- |

After rearranging Eqn A7, we get the integral equation:

|  | $-\int_{S}^{\infty} K\left( \hat{r},\hat{r}^{'} \right)d\hat{r}'=\left( 1-P_{\mathrm{bind}} \right)\int_{0}^{1} K\left( \hat{r},\hat{r}^{'} \right)g\left( \hat{r}^{'} \right)d\hat{r}'$  $+ \frac{P_{\lambda}}{\alpha^{2}}K\left( \hat{r},\alpha\right)\int_{0}^{1} g\left( \hat{r}^{'} \right){\hat{r}^{'}}^{2}d\hat{r}^{'}+ \int_{1}^{S} K\left( \hat{r},\hat{r}^{'} \right)g\left( \hat{r}' \right)d\hat{r}' - g\left( \hat{r} \right)$ | Eqn A8 |
| --- | --- | --- |

The Left-Hand Side of Eqn A8 can be solved analytically to yield the following function of $\hat{r}$:

|  | $F\left( \hat{r} \right)= -\int_{S}^{\infty} K\left( \hat{r},\hat{r}^{'} \right)d\hat{r}'$  $= \frac{1}{2}\left[ \text{erf}\left( \frac{S-\hat{r}}{\gamma\sqrt{2}} \right)+\text{erf}\left( \frac{S+\hat{r}}{\gamma\sqrt{2}} \right) \right]-1-\frac{\gamma^{2}}{S}K\left( \hat{r},S \right)$ | Eqn A9 |
| --- | --- | --- |

For the Right-Hand Side of Eqn A8, the integrals are numerically evaluated using the quadrature method. That is, the integral is approximated with a polynomial, which can be written as a weighted sum (Eqn A11). We discretize the domains $\hat{r}\in[0,1]$ and $\hat{r}^{'}\in[0,1]$ at the Chebyshev nodes with *n* = 20 (Eqn A10). By fitting the integrals at the Chebyshev nodes, we minimize the numerical error near the bounds of the integral.

|  | $\hat{r}_{i}=\frac{1}{2}+\frac{1}{2}\cos\left( \frac{2i-1}{2n}\pi\right)$ | Eqn A10 |
| --- | --- | --- |
|  | $\int_{0}^{1} f\left( \hat{r}^{'} \right)=\sum_{i=0}^{n-1} a_{i} f\left( \hat{r}_{i}^{'} \right)$ | Eqn A11 |

The coefficients, $\vec{a}$, are calculated from fitting the integral with the *n*^th^ degree polynomial:

|  | $\boldsymbol{A*}\vec{a} = \vec{h}$ | Eqn A12 |
| --- | --- | --- |
| where, | $\boldsymbol{A}_{i,j}=\left( \hat{r}_{j}^{'} \right)^{i}$  $h_{i}=\int_{0}^{1} \left( x \right)^{i}=\frac{1}{i+1}$ |  |
| for | $i,j= 0\ldots n-1$ |  |

Eqns A11 & A12 solve the first two terms of Eqn A8. To solve the last integral term (Eqn A14), the domains $\hat{r}\in[1,S]$ and $\hat{r}^{'}\in[1,S]$ are discretized at the Chebyshev nodes with *m* = 13 (Eqn A13).

|  | $\hat{r}_{j}=\frac{1+S}{2}+\frac{S-1}{2}\cos\left( \frac{2j-1}{2m}\pi\right)$ | Eqn A13 |
| --- | --- | --- |
|  | $\int_{1}^{S} f\left( \hat{r}^{'} \right)=\sum_{j=0}^{m-1} b_{j} f\left( \hat{r}_{j}^{'} \right)$ | Eqn A14 |

The coefficients, $\vec{b}$, are calculated by fitting the integral with the *m*^th^ degree polynomial:

|  | $\boldsymbol{B*}\vec{b} = \vec{h}$ | Eqn A15 |
| --- | --- | --- |
| where, | $\boldsymbol{B}_{i,j}=\left( \hat{r}_{j}^{'} \right)^{i}$  $h_{i}=\int_{1}^{S} \left( x \right)^{i}=\frac{{(S)}^{i+1} - 1}{i+1}$ |  |
| for | $i,j= 0 \ldots m-1$ |  |

Using Eqns A11, A12, A14 & A15, the Right-Hand Side of Eqn A8 is reduced to a function of $\hat{r}$ (Eqn A16). Because $\hat{r}$ is discretized the same as $\hat{r}^{'}$, Eqn A16 produces a system of equations, which can be written in matrix form (Eqn A17).

|  | $\left( 1-P_{\mathrm{bind}} \right)\int_{0}^{1} K\left( \hat{r},\hat{r}^{'} \right)g\left( \hat{r}' \right)d\hat{r}' + \frac{P_{\mathrm{bind}}}{\alpha^{2}}K\left( \hat{r},\alpha\right)\int_{0}^{1} g\left( \hat{r}^{'} \right){\hat{r}^{'}}^{2}d\hat{r}^{'} + \int_{1}^{S} K\left( \hat{r},\hat{r}^{'} \right)g\left( \hat{r}' \right)d\hat{r}' - g\left( \hat{r} \right)$  $=\left( 1-P_{\lambda} \right)\sum_{i=0}^{n-1} a_{i} K\left( \hat{r},\hat{r}_{i}^{'} \right)g\left( \hat{r}_{i}^{'} \right) +\frac{P_{\lambda}}{\alpha^{2}}K\left( \hat{r},\alpha\right)\sum_{i=0}^{n-1} a_{i} g\left( \hat{r}_{i}^{'} \right) {\hat{r}_{i}^{'}}^{2}+ \sum_{j=0}^{m-1} b_{j} K\left( \hat{r},\hat{r}_{j}^{'} \right)g\left( \hat{r}_{j}^{'} \right) - g\left( \hat{r} \right)$ | Eqn A16 |
| --- | --- | --- |
|  | $\vec{F}\boldsymbol{=M}\vec{g}$ | Eqn A17 |

$\vec{F}$ and $\vec{g}$ are, respectively, (n + m) × 1 column vectors of $F\left( \hat{r} \right)$ (Eqn A9) and $g\left( \hat{r} \right)$ evaluated at the discrete $\hat{r}$ points (Eqns A10 & A13). ***M*** is a (n + m) × (n + m) square matrix, built from factoring out $g\left( \hat{r} \right)$ and $g\left( \hat{r}^{'} \right)$ from Eqn A16.

Hence the technique is to first guess a value for P_bind_. Based on this guess, we approximate $g\left( \hat{r} \right)$ by solving Eqn A17. This approximation is sufficient to evaluate Eqn A3 and determine the accuracy of the guess. If Eqn A3 is incorrect, then we use the Secant Method to update the guess for P_bind_. This process is repeated until the error falls below a threshold (10^-15^). We find P_bind_ ≈ 0.001.

One important caveat for our system is to double the final value of P_bind_ as obtained using the above technique. The above equations were derived for a system in which both reactant species are in solution. Accordingly, ligand molecules can approach a receptor from any direction. However, in our system, ligand molecules can only approach a receptor from half as many directions (**Fig A1**). We double the probability and use P_bind_ = 0.002 in our simulations. This adjustment was also made in a recently published model [2].

In the language of the $\lambda-\bar{\varrho}$ method, we adjust Eqn A3, which integrates over spherical surface areas, to integrate over hemispherical surface areas (Eqn A18).

|  | $\kappa- P_{\mathrm{bind}}\int_{0}^{1} 2\pi z^{2}g\left( z \right)dz = 0$ | Eqn A18 |
| --- | --- | --- |


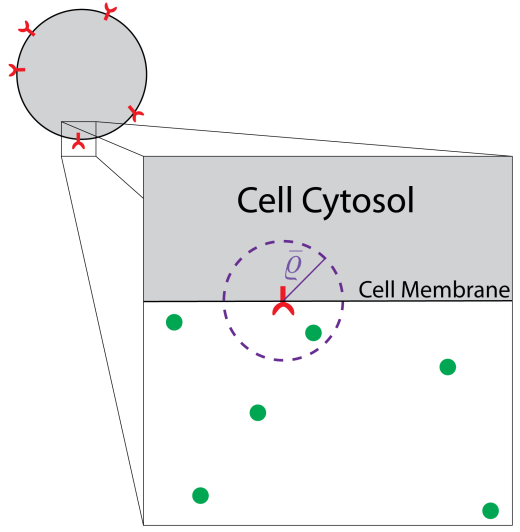


**Figure A1: Rationale for Doubling P_bind_**

In the upper left corner, we show a 2D cartoon of the cell in our simulation: a circle with unbound Ste2 receptors on the surface. By focusing closely on a single receptor, we see how the scale of the binding radius, r_bind_, compares to the scale of the cell. That is, the membrane appears as a straight line, because the cell’s radius (2.5μm) is three orders of magnitude larger than the binding radius (4nm). The membrane divides the binding volume (purple circle) exactly in half. Pheromone molecules can only enter this volume from one half.

***References***

1. Lipková J, Zygalakis KC, Chapman SJ, Erban R (2011) Analysis of Brownian Dynamics Simulations of Reversible Bimolecular Reactions. SIAM J Appl Math 71: 714–730.

2. Dobramysl U, Rüdiger S, Erban R (2016) Particle-based Multiscale Modeling of Calcium Puff Dynamics. Multiscale Model Simul 14: 997–1016.

***B. Calculating Pheromone Reflection off the Cell Surface***

The cell membrane is impermeable to pheromone. Our model prevents pheromone from diffusing into the cell by reflecting molecules off the surface of the cell. In our simulations, the cell is a sphere with a radius of *R* and center at (0, 0, 0). **Fig B1** shows how we calculate these reflections.


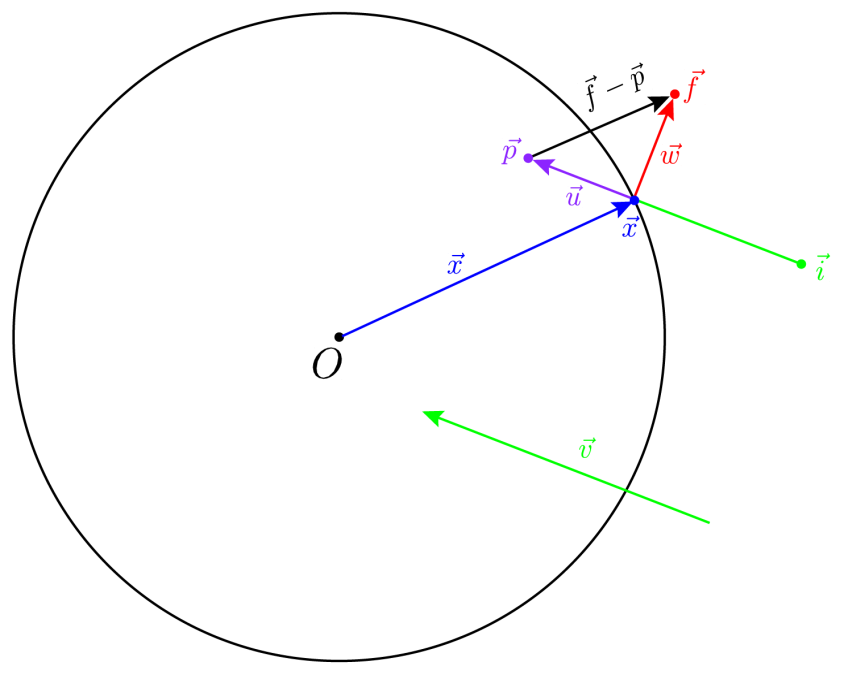


**Figure B1: Reflecting Pheromone off Cell Surface**

A diagram of important vectors used to calculate how a pheromone molecule reflects off the surface of the cell during a single diffusion step. The molecule is initially at $i$, and after freely diffusing for one step, is positioned inside the cell at $p$. The desired, reflected position is $f$. Although the simulation is in 3D and the cell is modeled as a sphere, all of these points and vectors lie on a single plane (defined by the three points *i*, *p* and *O*). By using vector algebra, we may solve the system on a plane and the resulting equations will be valid in 3D.

Let $i$ be the initial position of the pheromone molecule before diffusion. Let $p$ be the “proposed” position after diffusion, which is undesirably located inside the cell. The vector $v$ is the attempted trajectory of the pheromone molecule for a single time step. That is, $v=p-i$. If the molecule reflected off the cell surface, then its final position would be$f$. Hence, we wish to find a set of equations to calculate $f$, given $i$, $p$ and the dimension of the sphere.

First, we calculate the position where the molecule contacts the cell surface: $x$. The attempted trajectory, $v$, defines a line which can be written as $i+tv$. The intersection, $x$, lies on this line:

|  | $x=i+t_{x}v$ | Eqn B1 |
| --- | --- | --- |

Knowing that $x \cdot x = R^{2}$, we can solve for $t_{x}$:

|  | $\left( i+t_{x}v \right)\cdot\left( i+t_{x}v \right)=R^{2}$ | Eqn B2 |
| --- | --- | --- |
|  | $t_{x}=\frac{-v\cdot i - \sqrt{\mathbf{Det}}}{\left\Vert v \right\Vert^{2}}$ | Eqn B3 |
| where, | $\mathbf{Det}=\left( v\cdot i \right)^{2}+\left( \left\Vert v \right\Vert^{2} \right)\left( R^{2}-\left\Vert i \right\Vert^{2} \right)$ |  |

Second, we calculate the vector $u$ (Eqn B4). This vector is the portion of the molecule’s trajectory that incorrectly traveled into the cell. Third, to correct the trajectory, we calculate $w$, which is equivalent to $u$ except that they have opposite radial components. The direction pointing away from the cell is calculable from the intersection point: $\hat{x}=\frac{x}{\left\| x \right\|}=\frac{x}{R}$. Eqn B5 shows that $w$ and $u$ differ by twice their radial components.

|  | $u=p-x$ | Eqn B4 |
| --- | --- | --- |
|  | $w-u=2\left( - \left( u\cdot\hat{x} \right) \right)\hat{x}$  $w-u=-\left( \frac{2}{R^{2}} \right)\left( u\cdot x \right)\left( x \right)$ | Eqn B5 |

Finally, because $w-u=f-p$, we can calculate $f$:

|  | $f=p-\left( \frac{2}{R^{2}} \right)\left( u\cdot x \right)\left( x \right)$ | Eqn B6 |
| --- | --- | --- |

Numerically, we calculate $x$ from Eqns B3 & B1 using known values for $i$, $p$ and *R*. We then calculate $u$ from Eqn B4. Lastly, we find the final reflected position, $f$, by solving Eqn B6.

***C. Injection Boundary Condition: Derivation of Eqns 5 – 8***

We simulate a linear pheromone gradient along the x-axis, which requires special boundary conditions at the x-boundaries defined by the planes: $x=\pm\frac{L}{2}$. We must define how individual molecules behave at these boundaries.

*C.1 Gradient Method 1*

One method to create a pheromone gradient is to fix the pheromone concentration at each x-boundary. The fixed concentrations are molecular reservoirs in the semi-infinite domains ($x<-\frac{L}{2}$ and $x>\frac{L}{2}$) that extend past each planar boundary. A molecular reservoir, analogous to a thermal reservoir, is a large enough volume such that adding or removing molecules does not appreciably change the concentration. Molecules are exchanged between the simulation volume and the reservoir in processes called ‘injection’ (entering the simulation volume) and ‘ejection’ (exiting the simulation volume). These two processes are independent. For ejection, the molecules are considered part of the reservoir and are no longer simulated. For injection, new molecules diffuse into the simulation volume. Here, we derive how many molecules to inject and where to inject them.

For simplicity, we shift the simulation domain such that $x\in\left[ 0,L \right]$ and consider the x=0 boundary. Given the molecules’ diffusion constant, *D*, the concentration of the reservoir, *c*, the time step Δt, and the area of the boundary, *a*, we derive how many molecules will enter the simulation volume from the reservoir. The probability distribution at time Δt for a molecule starting at 0 is:

|  | $P\left( \tilde{x} \right)=\frac{1}{\sqrt{4\pi D\Delta t}}\exp\left[ -\frac{\tilde{x}^{2}}{4D\Delta t} \right]$ | Eqn C1.1 |
| --- | --- | --- |


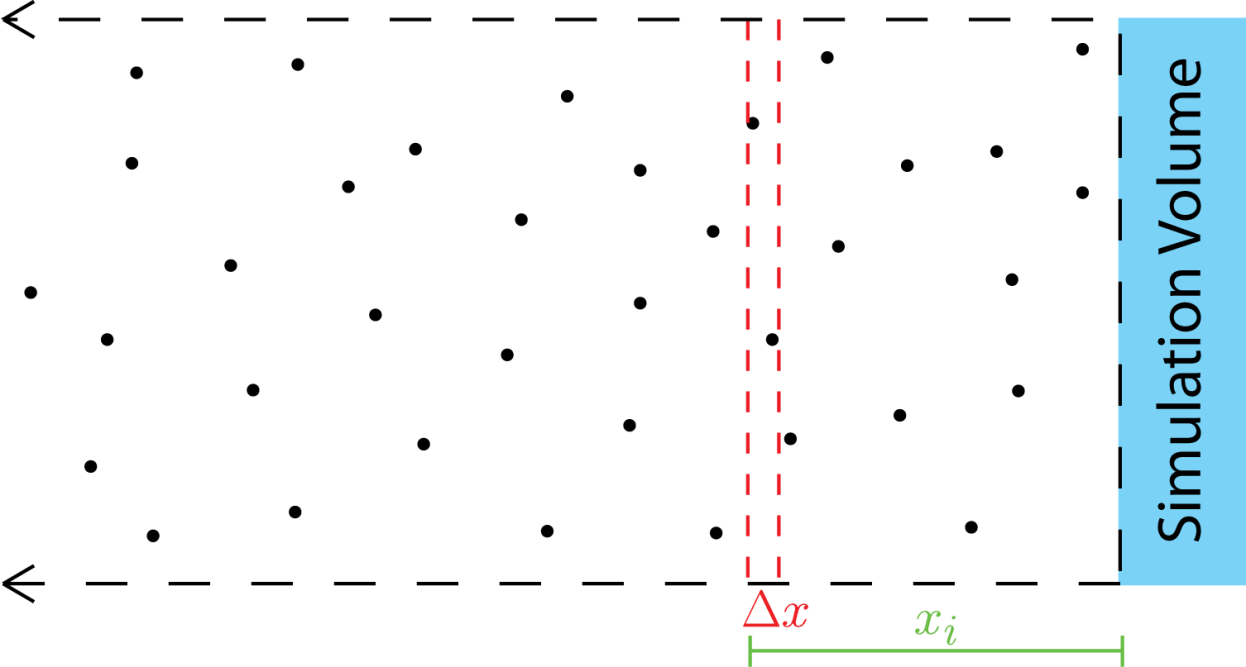


**Figure C1: Molecular Reservoir**

A cartoon of the molecular reservoir outside one boundary. The simulation volume is colored in blue and continues finitely off the page. The molecular reservoir has a white background and extends to –∞. The border is indicated by the black, vertical dashed line. Also shown is a slice of the reservoir, with thickness Δx, at a distance x_i_ from the boundary.

Consider a thin slice of the semi-infinite volume, a distance x_i_ from the boundary (**Fig C1**). To end up in the simulation domain the particle must diffuse a distance of at least x_i_ and less than *L* + x_i_. The probability is given by:

|  | $P_{\mathrm{inj}}\left( x_{i} \right)=\int_{x_{i}}^{L+x_{i}} P\left( \tilde{x} \right)d\tilde{x}=\frac{1}{2}\left[ 1-\mathrm{erf}\left( \frac{x_{i}}{\sqrt{4D\Delta t}} \right) \right]$ | Eqn C1.2 |
| --- | --- | --- |

The integral’s simplification takes advantage of the approximation $\mathrm{erf}\left( \frac{L+x_{i}}{\sqrt{4D\Delta t}} \right)=1$, because $L\gg\sqrt{4D\Delta t}$ for our values (**Table 1**). Note that for the slice located at the boundary, x_0_ = 0, P_inj_ = ½ as expected; a molecule at x = 0 has an equal chance of diffusing right (into the volume) or left (remaining in the reservoir). The entire reservoir can be divided into adjacent, thin slices. For the *i*^th^ slice, the number of molecules injected is given by *N* · P_inj_(x_i_), where *N* is the number of molecules in this slice. Because the reservoir has a fixed concentration of molecules, *N* = *c* · a · Δx. We sum the contribution from each slice and get a total number of injected molecules:

|  | $n_{\mathrm{inj}}=c\cdot a\sum_{i=0}^{\infty} P_{\mathrm{inj}}\left( x_{i} \right)\Delta x$ | Eqn C1.3 |
| --- | --- | --- |

We make the slices infinitely small (Δx → 0), such that Eqn C1.3 becomes a solvable integral:

|  | $n_{\mathrm{inj}}=c\cdot a\int_{0}^{\infty} P_{\mathrm{inj}}\left( x \right)dx=c\cdot a\sqrt{\frac{D\Delta t}{\pi}}$ | Eqn C1.4 |
| --- | --- | --- |

*C.2 Injection Distances*

We define the injection distance, d_inj_, as the distance from the boundary that a newly injected molecule is placed. We derive the probability of a molecule being injected a distance d_inj_. For example, **Fig C2** shows a molecule can diffuse to the d_inj_ position in many ways. That is, the molecule has many starting positions, from x = 0 (at the boundary) to x → –∞. Let Δx be the distance the molecule diffuses; therefore, $\Delta x\in\left[ d_{\mathrm{inj}} \right.,\left. \infty\right)$.


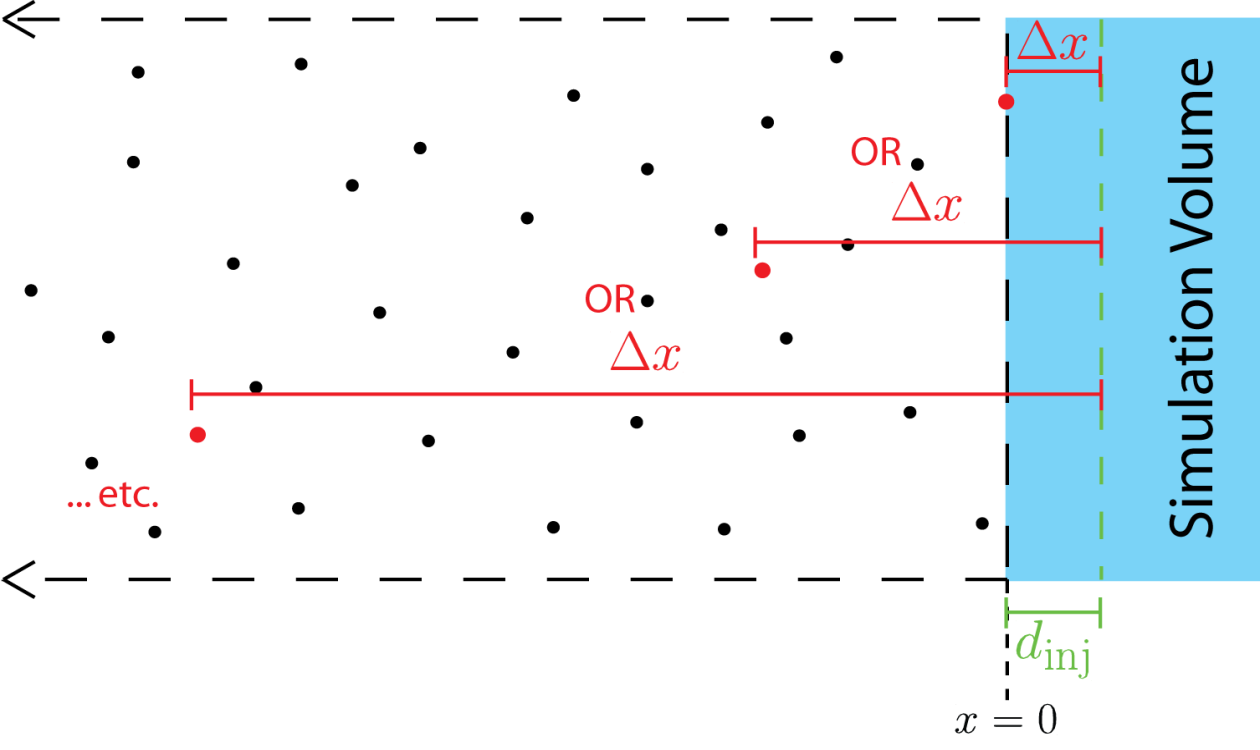


**Figure C2: Injection Distance**

A cartoon showing the many ways a molecule could diffuse a fixed distance (d_inj_) into the simulation volume (blue). The red dots show various starting positions; the molecule must travel a distance Δx in order to end at d_inj_.

The probability of diffusing d_inj_ into the simulation volume is the sum of the probabilities from each potential starting position (Eqn C2.1). We then normalize this distribution to get the Probability Density Function (Eqn C2.2).

|  | $P\left( d_{\mathrm{inj}} \right)=\int_{d_{\mathrm{inj}}}^{\infty} P\left( \Delta x \right) d\left( \Delta x \right)=\frac{1}{2}\left[ 1-\mathrm{erf}\left( \frac{d_{\mathrm{inj}}}{\sqrt{4D\Delta t}} \right) \right]$ | Eqn C2.1 |
| --- | --- | --- |
|  | $PDF\left( d_{\mathrm{inj}} \right)=\sqrt{\frac{\pi}{4D\Delta t}}\left[ 1-\mathrm{erf} \left( \frac{d_{\mathrm{inj}}}{\sqrt{4D\Delta t}} \right) \right]$ | Eqn C2.2 |

Note that the distribution depends only on the diffusion constant and the time step. We empirically verify this distribution (Eqn C2.2). We simulate diffusion in a cubic volume and record the distance the particles traveled beyond the boundaries in one time step. **Fig C3** shows the theoretical and empirical distributions. The recorded list has over 2.5 million values, and we draw a d_inj_ for each new molecule during the simulation.


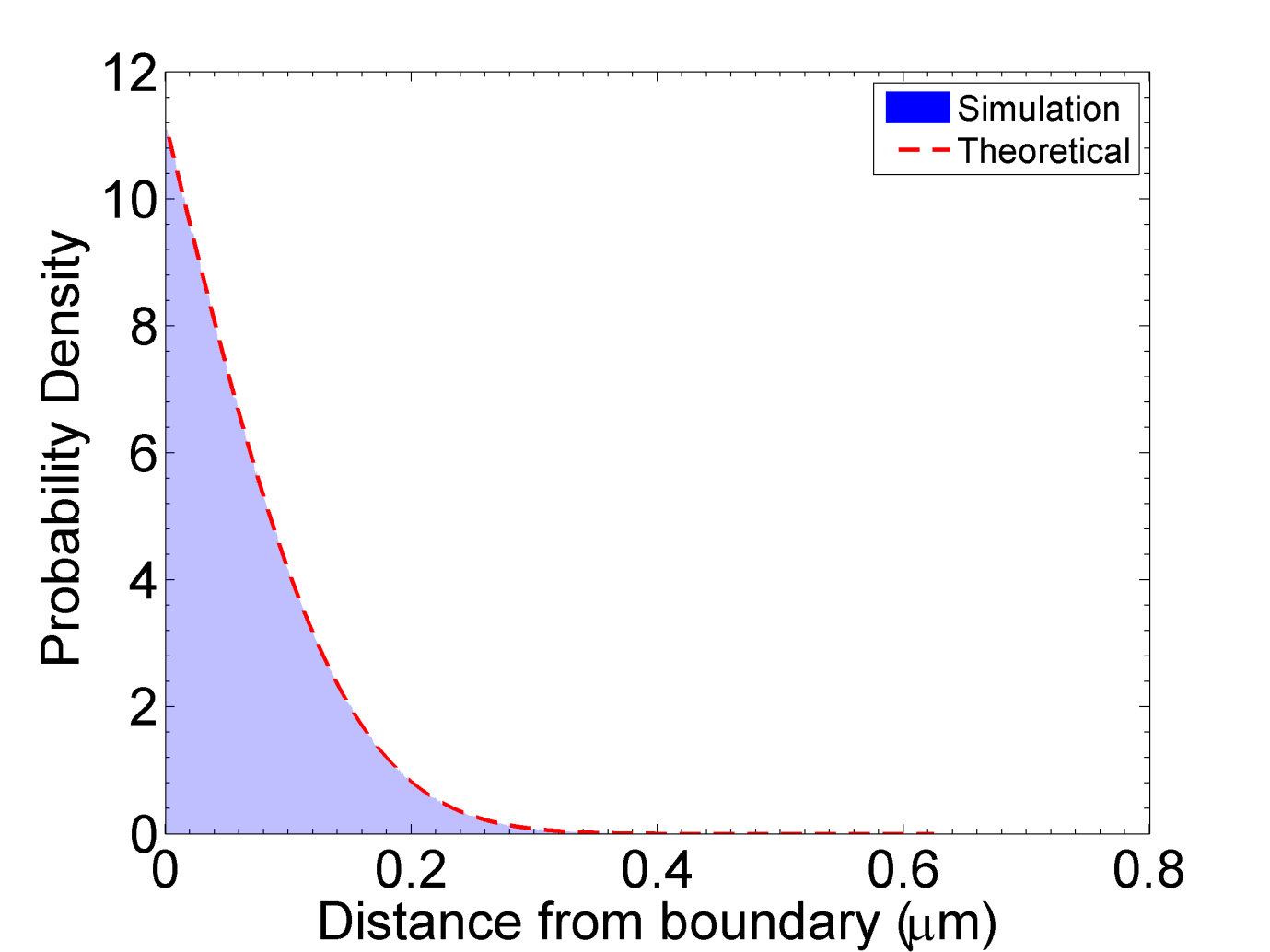


**Figure C3: Probability Density of Injection Distance**

Here we plot the probability density for an injected molecule to be placed a distance d_inj_ into the simulation volume. The dashed red line is the theoretical, normalized probability density function (Eqn C2.2). The blue histogram is from simulated data. In this case, $D=125\frac{{\mu m}^{2}}{s}$ and Δt = 50μs.

Ideally, our injection algorithm would generate a random injection distance from this distribution during the simulation (Eqn C2.2). However, the corresponding Cumulative Distribution Function:

|  | $CDF\left( d_{\mathrm{inj}} \right)=\int_{0}^{d_{\mathrm{inj}}} PDF\left( {d_{\mathrm{inj}}}^{'} \right) d\left( {d_{\mathrm{inj}}}^{'} \right)$  $=\sqrt{\frac{\pi}{4D\Delta t}}d_{\mathrm{inj}}\left( 1-\mathrm{erf}\left( \frac{d_{\mathrm{inj}}}{\sqrt{4D\Delta t}} \right) \right)+1-\exp\left[ \frac{-{d_{\mathrm{inj}}}^{2}}{4D\Delta t} \right]$ | Eqn C2.3 |
| --- | --- | --- |

cannot be inverted. Instead, we draw randomly from the previously generated list of injection distances. This table-lookup method is computationally efficient.

*C.3 Gradient Method 2*

In Section C.1, we describe a method to establish a linear gradient by fixing the concentration at the x-boundaries. A second method is to flow molecules from one x-boundary and partially absorb molecules at the opposing boundary. As in Section C.1, we shift the simulation domain such that $x\in\left[ 0,L \right]$. New molecules are added to the system at the x = L boundary, which has a higher concentration than at x = 0.

First, we determine the injection rate at the x = L boundary. We define the desired gradient, *g*, and the concentration at x = L is c_L_. Eqn C3.1 describes the desired concentration profile at this boundary: $\tilde{x}=x-L$.

|  | $c\left( \tilde{x} \right)=g\tilde{x}+c_{L}$ | Eqn C3.1 |
| --- | --- | --- |

The gradient is positive (*g* > 0). We calculate the number to inject by changing $c=c\left( \tilde{x} \right)$ in Eqn C1.3. The resulting discrete sum is converted to an integral and solved:

|  | $n_{\mathrm{inj}}=a\int_{0}^{\infty} c\left( \tilde{x} \right)P_{\mathrm{inj}}\left( \tilde{x} \right)d\tilde{x}=\frac{1}{2}a\cdot g\cdot D\Delta t+ c_{L}\cdot a\sqrt{\frac{D\Delta t}{\pi}}$ | Eqn C3.2 |
| --- | --- | --- |

Second, we determine the absorption probability at the x = 0 boundary. Eqn C3.3 describes the desired concentration profile at this boundary; c_0_ is the desired concentration at x = 0.

|  | $c\left( x \right)=gx+c_{0}$ | Eqn C3.3 |
| --- | --- | --- |

The absorption probability, P_Abs_, is the probability a molecule, which has diffused outside the boundary (x < 0), is removed from the simulation. If the molecule is not removed, then the molecule is reflected back into the simulation volume. The two quantities are related:

|  | $P_{\mathrm{Abs}}=1-P_{\mathrm{Ref}}$ | Eqn C3.4 |
| --- | --- | --- |

The reflection probability, P_Ref_, can be calculated from the expected number of molecules exchanged between the reservoir and simulation volume:

|  | $P_{\mathrm{Ref}}=\frac{n_{\mathrm{in}}}{n_{\mathrm{out}}}$ | Eqn C3.5 |
| --- | --- | --- |

The expected number of molecules that exit the simulation volume is n_out­_, given by:

|  | $n_{\mathrm{out}}=a\int_{0}^{L} c\left( x \right)P_{\mathrm{inj}}\left( x \right)dx=\frac{1}{2}a\cdot g\cdot D\Delta t+ c_{0}\cdot a\sqrt{\frac{D\Delta t}{\pi}}$ | Eqn C3.6 |
| --- | --- | --- |

Eqn C3.6 is valid for $L\gg\sqrt{4D\Delta t}$, which is the case for our values (**Table 1**). The expected number of molecules that enter the simulation is n_in_. To calculate n_in_, a slight change in variables is helpful: x' = – x. Accordingly, the desired concentration profile (Eqn C3.3) changes:

|  | $c\left( x' \right)=-gx'+c_{0}$ | Eqn C3.7 |
| --- | --- | --- |

Because concentration can never be negative ($c\left( x^{'} \right)\geq0$), the domain of x' is limited: $x^{'}\leq\frac{c_{0}}{g}$. The general solution for n_in_ is:

|  | $n_{\mathrm{in}}=a\int_{0}^{\frac{c_{0}}{g}} c\left( x^{'} \right)P_{\mathrm{inj}}\left( x^{'} \right)dx^{'}$  $=\frac{1}{2}a\left[ c_{0}\sqrt{\frac{D\Delta t}{\pi}}\left( 2-e^{\frac{-{c_{0}}^{2}}{g^{2} \cdot4D\Delta t}} \right)+\frac{1}{2}\frac{{c_{0}}^{2}}{g}-\left( \frac{1}{2}\frac{{c_{0}}^{2}}{g}+gD\Delta t \right)\mathrm{erf}\left( \frac{c_{0}}{g\sqrt{4D\Delta t}} \right) \right]$ | Eqn C3.8 |
| --- | --- | --- |

For our values (Table 1), $\frac{c_{0}}{g}\gg\sqrt{4D\Delta t}$; therefore, Eqn C3.8 simplifies:

|  | $n_{\mathrm{inj}}=c_{0}\cdot a\sqrt{\frac{D\Delta t}{\pi}}-\frac{1}{2}a\cdot g\cdot D\Delta t$ | Eqn C3.9 |
| --- | --- | --- |

Substituting Eqns C3.6 & C3.9 into Eqn C3.5, we get the following expression for P_Ref_:

|  | $P_{\mathrm{Ref}}=1-\frac{g}{c\sqrt{\frac{1}{\pi D\Delta t}} + \frac{1}{2}g}$ | Eqn C3.10 |
| --- | --- | --- |

***D. Receptor Diffusion on the Cell Surface: Derivation of Eqn 9***

To calculate receptor diffusion on the surface of the cell, we first diffuse the receptor freely in 3D, and second, we project that new position onto the surface of the cell. Eqn 9 describes the latter step. Let $(\tilde{x}, \tilde{y}, \tilde{z})$ be the diffused position, which we want to project onto a sphere with a radius of *R* and center at (0, 0, 0). We can write this position in spherical coordinates as $(\tilde{r}, \tilde{\theta}, \tilde{\phi})$, where $\tilde{r}=\sqrt{\tilde{x}^{2}+\tilde{y}^{2}+\tilde{z}^{2}}$, and $\tilde{\theta}$ & $\tilde{\phi}$ are spherical angles. To convert from spherical to Cartesian coordinates:

|  | $\tilde{x}=\tilde{r}\sin\tilde{\theta}\cos\tilde{\phi}$ | Eqn D1 |
| --- | --- | --- |
|  | $\tilde{y}=\tilde{r}\sin\tilde{\theta}\sin\tilde{\phi}$ | Eqn D2 |
|  | $\tilde{z}=\tilde{r}\cos\tilde{\theta}$ | Eqn D3 |

The final projected position, (x, y, z), has the same spherical angles $\tilde{\theta}$ and $\tilde{\phi}$ but a radius of *R*. Because the angles are the same, we can equate the trigonometric factors as follows.

|  | $\frac{\tilde{x}}{\tilde{r}}=\sin\tilde{\theta}\cos\tilde{\phi}=\frac{x}{R}$ | Eqn D4 |
| --- | --- | --- |
|  | $\frac{\tilde{y}}{\tilde{r}}=\sin\tilde{\theta}\sin\tilde{\phi}=\frac{y}{R}$ | Eqn D5 |
|  | $\frac{\tilde{z}}{\tilde{r}}=\cos\tilde{\theta}=\frac{z}{R}$ | Eqn D6 |

Multiplying Eqns D4 – D6 by *R*, yields Eqns 9b – 9d.

***E. Measuring the Pheromone Distribution***

To determine the distribution of pheromone molecules, we discretize the simulation volume with thick-walled cylinders (**Fig E1**). The naïve approach is to discretize the space with small cubes; however, we recognize that our system is symmetric in the ‘y’ and ‘z’ dimensions. Therefore, we reduce these two spatial dimensions into one radial dimension: $\rho=\sqrt{y^{2}+z^{2}}$. We discretize x-axis using bins of width Δx and discretize the radial dimension using bins of width Δρ. Each pheromone molecule is tallied according to its x-position and distance from the x-axis ($\sqrt{y^{2}+z^{2}}$). We determine the average number of pheromone molecules in each bin and subsequently calculate the concentration by dividing by the volume of each cylinder. The pheromone concentration is a function of *x* and ρ: c = c(x, ρ). Note that for ρ ≤ 2.5µm and $x\in\left[ -2.5, 2.5 \right] \mu m$, the cylindrical bin intersects with, or lies inside the cell. Because pheromone is excluded from the cell’s interior, the effective volume of these bins is reduced. The concentration profiles presented in the main text are c(x) for ρ ≈ 1.01µm. Hence, for –2.28µm ≤ *x* ≤ 2.28µm, the expected and measured pheromone concentration is 0 nM.


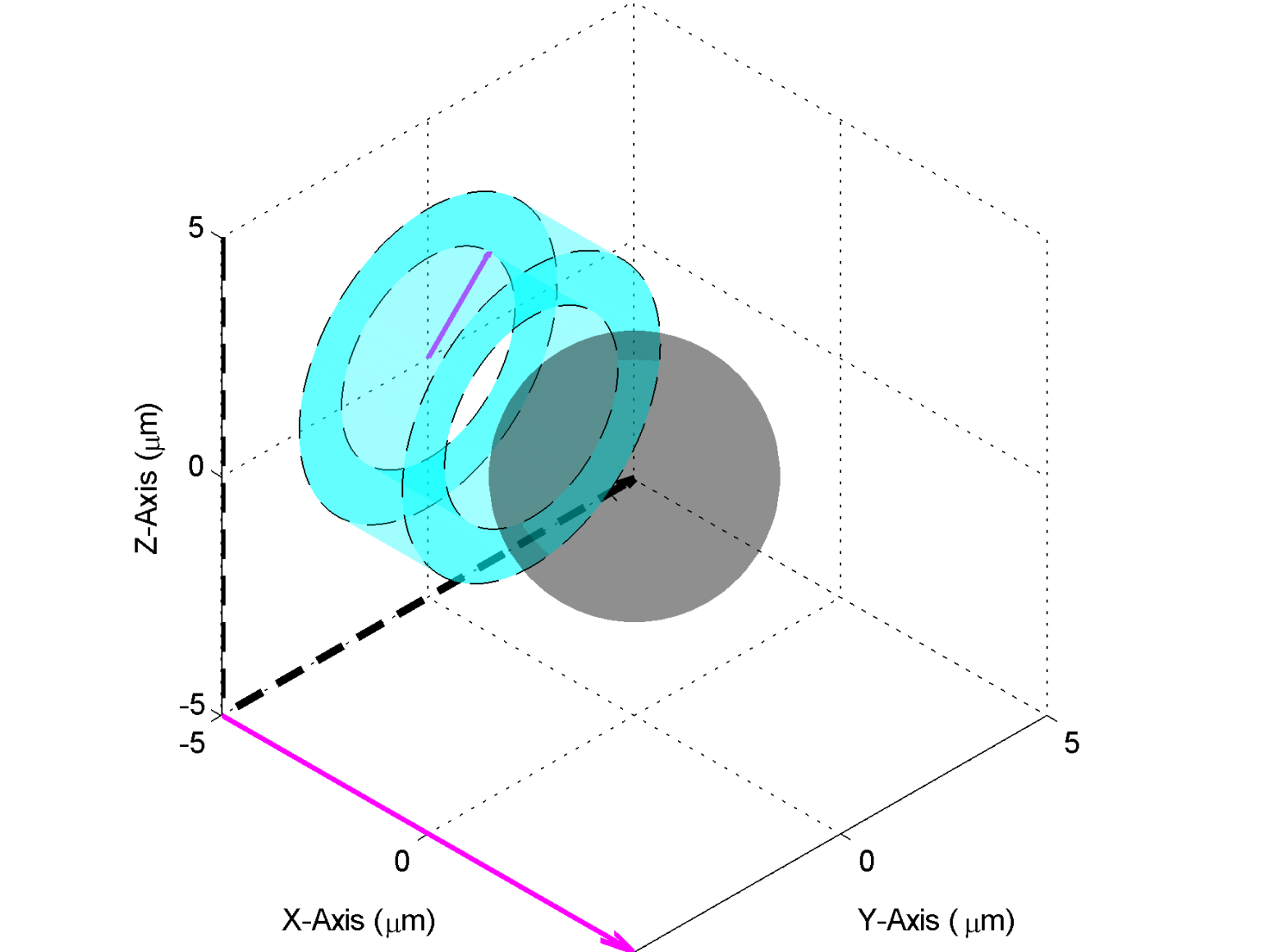


**Figure E1: Cylindrical Bins**

This cartoon depicts how a single cylindrical bin is oriented with respect to the simulation volume. The cell is shown as a gray sphere in the center of the volume. The height of the cylinder is Δx, and the thickness of the wall is Δρ. We count the average number of pheromone molecules between the walls of this cylinder and divide by the volume to estimate the concentration. Specifically, we choose $\Delta x=\frac{10\mu m}{64}=0.15625\mu m$ and $\Delta\rho=\frac{5\mu m}{32}=0.15625\mu m$. Molecules located near the edges of the simulation volume, ρ > 5µm, are not tallied.
