# Supplementary material for: Testing the limits of gradient sensing
Source: PLoS Comput Biol. 2017 Feb 16;13(2):e1005386. doi: 10.1371/journal.pcbi.1005386 (PMC5347372; doi:10.1371/journal.pcbi.1005386)
Supplement: S2 Text — This text contains some additional supporting results, including gradient sharpening in a larger simulation volume (Section A), gradient sharpening in simulations with “slow” reaction rates (Section B), receptor occupancies in the front and back halves of the cell in a shallow gradient (Section C), a negative control for our confidence measure (Section D) and results from simulations with smaller cells of radius 1.75μm (Section E). (PDF) [file pcbi.1005386.s002.pdf]

“Testing the Theoretical Limits of Gradient Sensing in Yeast”

V Lakhani and T Elston

Text S2: Supplemental Results

A. Gradient Sharpening in Large Volume 2
B. Gradient Sharpening for “slow” Reaction Rates 3
C. Front & Back Occupancy Distributions in 0.1 nM/µm 4
D. Confidence Measure in Uniform Pheromone 5
E. Results from Small Cells (r = 1.75µm) 6

A. Gradient Sharpening in Large Volume

The same gradient sharpening effects are seen when the simulation volume is increased. The figure below shows the results when a simulation volume of 15 × 15 × 15 μm^3 is used. The results are the same as in Fig. 4 of the main text, which are for a volume of 10 × 10 × 10 μm^3.

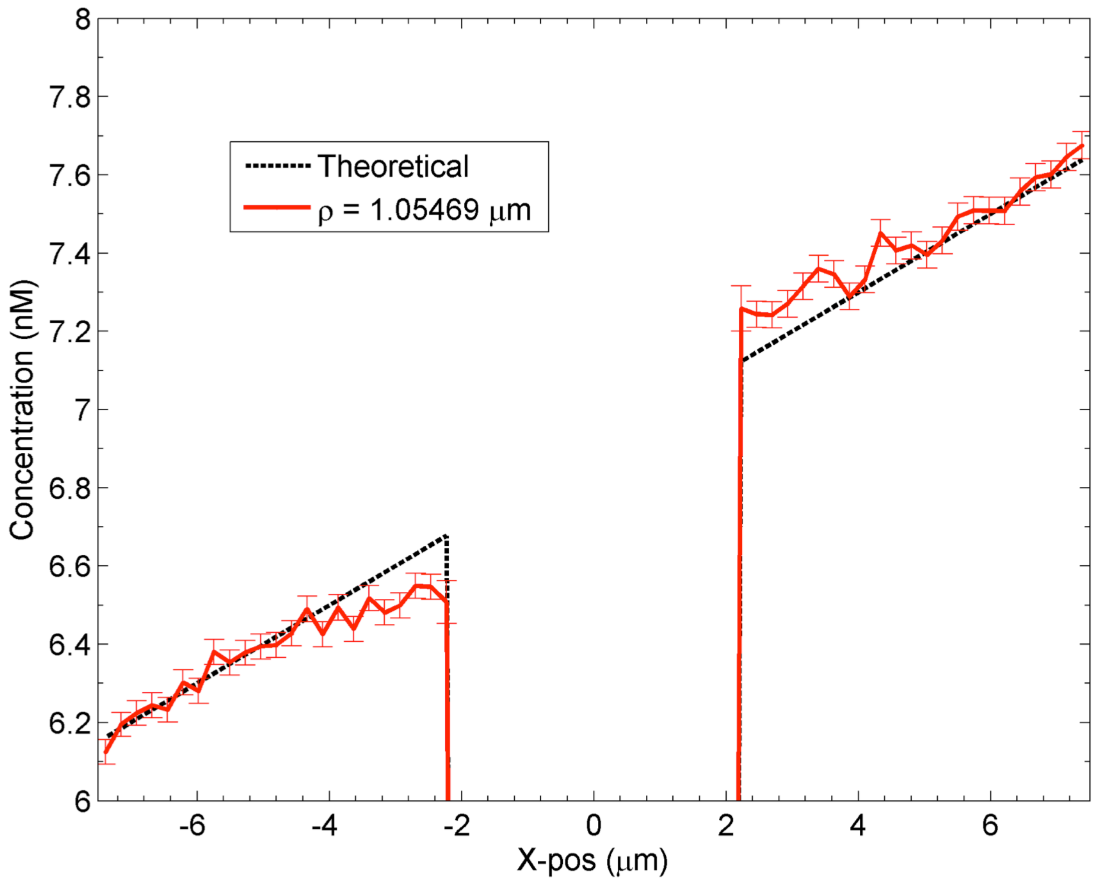

Figure A1: Gradient Sharpening
We plot the pheromone concentration as a function of *x*, with $\rho = 1.05 \mu m$. The red curve is calculated from an average of eight simulations, which include binding and unbinding reactions ($k_{on} = 1.6 \times 10^6$ (M·s)$^{-1}$ and $k_{off} = 1.1 \times 10^{-2}$ 1/s).

B. Gradient Sharpening for "slow" Reaction Rates

Simulations with "slow" reaction rates also show the same gradient sharpening effect. The figure below shows results from simulations similar to Fig 4 except that the reaction rates are ten times slower: $k_{on} = 1.6\times10^5\ (M\cdot s)^{-1}$ and $k_{off} = 1.1\times10^{-3}$ 1/s.

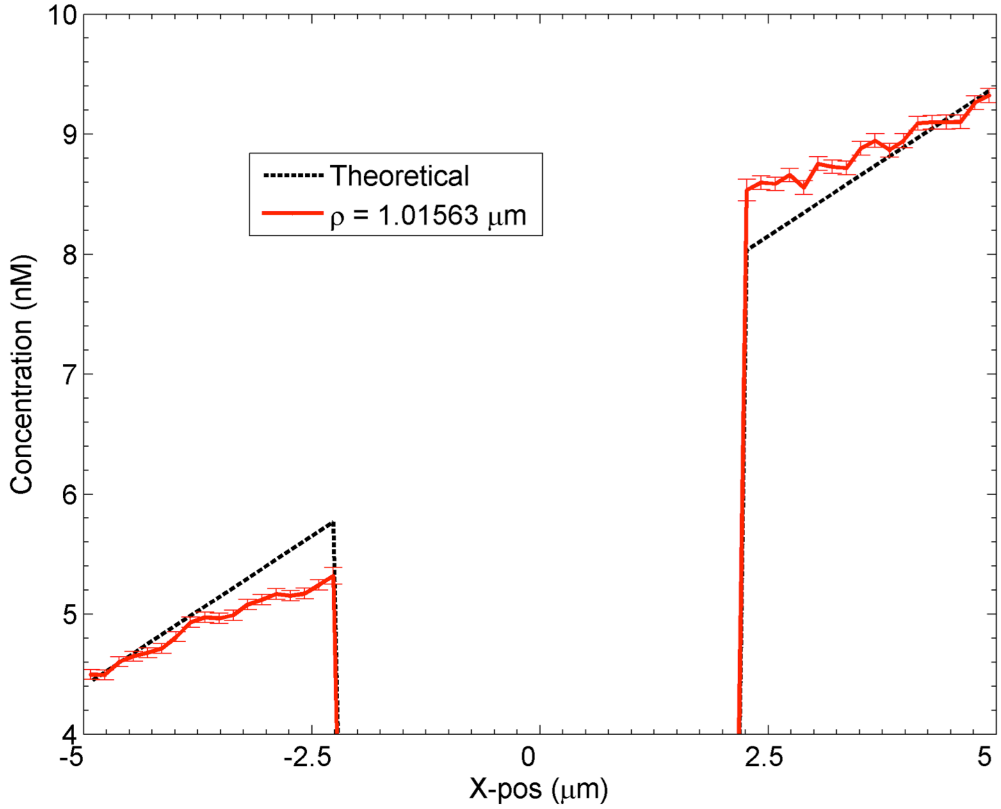

Figure B1: Gradient Sharpening
We plot the pheromone concentration as a function of x, with $\rho\approx1.02\mu m$. The red curve is calculated from an average of eight simulations, which include binding and unbinding reactions ($k_{on} = 1.6\times10^5\ (M\cdot s)^{-1}$ and $k_{off} = 1.1\times10^{-3}$ 1/s). This figure corresponds to the same simulations shown in Fig 5A.

C. Front & Back Occupancy Distributions in 0.1 nM/μm

Simulations in shallow gradients (0.1 nM/μm) show a larger overlap region in the distribution of receptor occupancy in the front and back halves of the cell. This figure is similar to Fig 5A except the gradient is shallower.

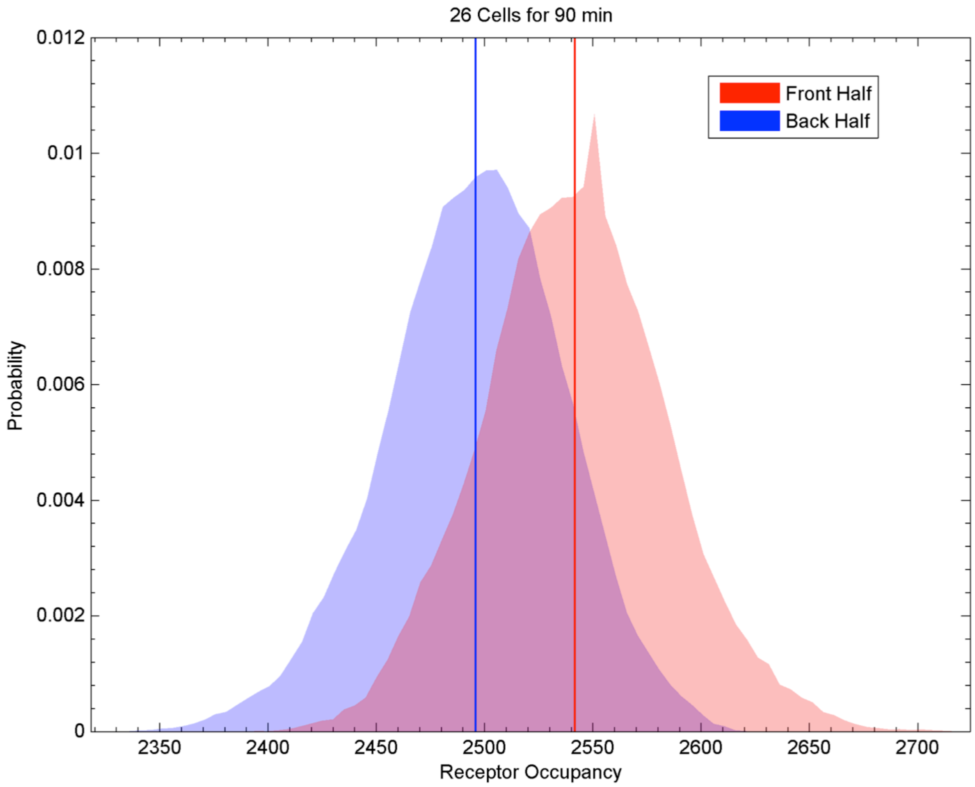

Figure C1: Receptor Occupancy in Shallow Gradient
Histograms show the number of occupied receptors in the front (red) and back (blue) halves of the cell. These plots are created from compiling twenty-six simulations in a shallow gradient (0.1 nM/μm) and "slow" reaction rates: $k_{on} = 1.6\times10^5$ $(M\cdot s)^{-1}$ and $k_{off} = 1.1\times10^{-3}$ 1/s.

D. Confidence Measure in Uniform Pheromone

As a negative control, we calculated the confidence value (Eqn 9) for simulations in uniform pheromone concentration. This result is shown in the figure below. The reaction rates are "fast": $k_{on} = 1.6\times10^{6}\ (M\cdot s)^{-1}$ and $k_{off} = 1.1\times10^{-2}$ 1/s.

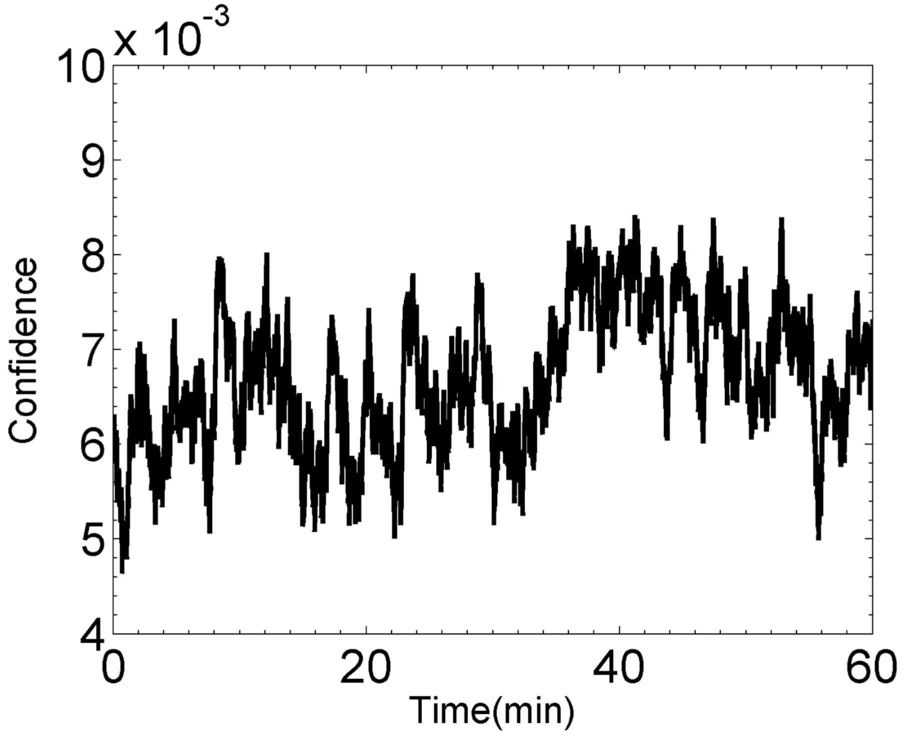

Figure D1: Confidence in Uniform Gradient
These confidence values are an average of sixteen simulations with "fast" reaction rates and no pheromone gradient. Averaging these values over all time gives a confidence ~ 6.7×10^{-3}. This value is lower than the values reported in Fig 10.

E. Results from Small Cells (r = 1.75μm)

Simulations with small cells show both gradient sharpening and overlap in receptor occupancy (between the front and back halves). The figures below showing these results are calculated from simulations with a steep gradient (0.5 nM/μm) and "fast" reaction rates: $k_{on} = 1.6\times10^6$ $(M\cdot s)^{-1}$ and $k_{off} = 1.1\times10^{-2}$ 1/s. The two figures are compiled from eight simulations.

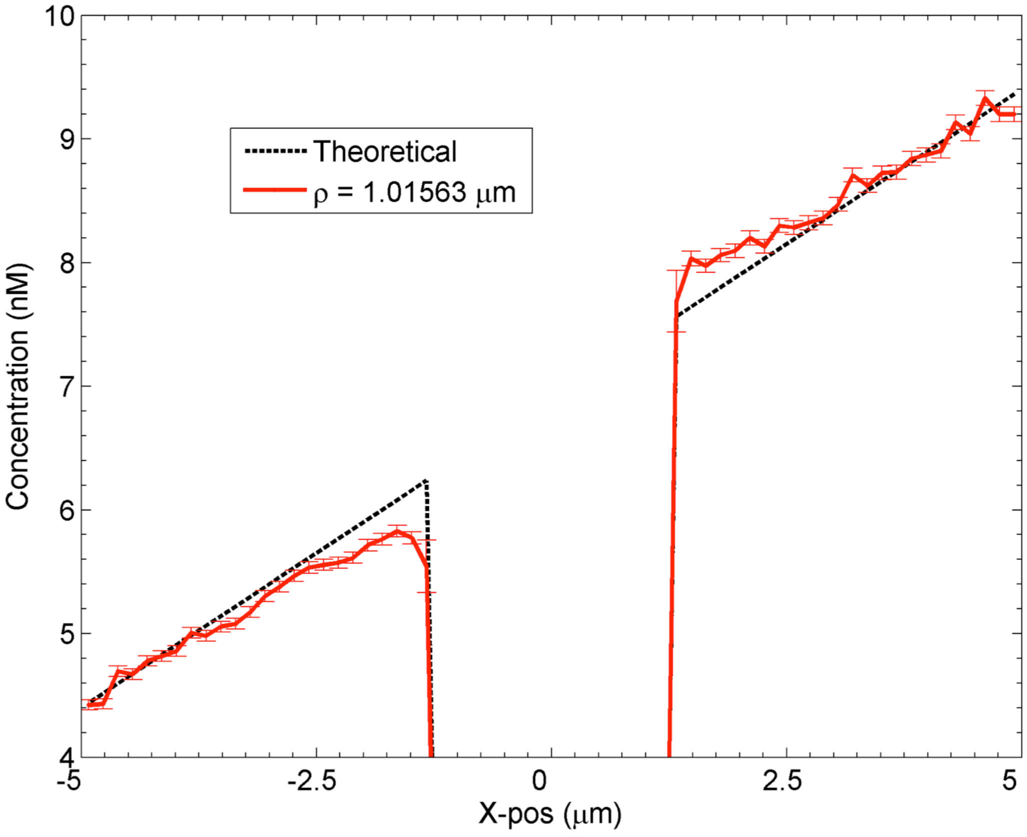

Figure E1: Gradient Sharpening
We plot the pheromone concentration as a function of *x*, with *ρ=1.05μm*. The red curve is calculated from an average of eight simulations, which include binding and unbinding reactions ($k_{on} = 1.6\times10^6$ $(M\cdot s)^{-1}$ and $k_{off} = 1.1\times10^{-2}$ 1/s). This figure is similar to Fig 4A except that the radius of the cell is smaller (1.75μm) than the simulations presented in the main text (2.5μm). Note that sharpening effect here is lesser than in Fig 4A.

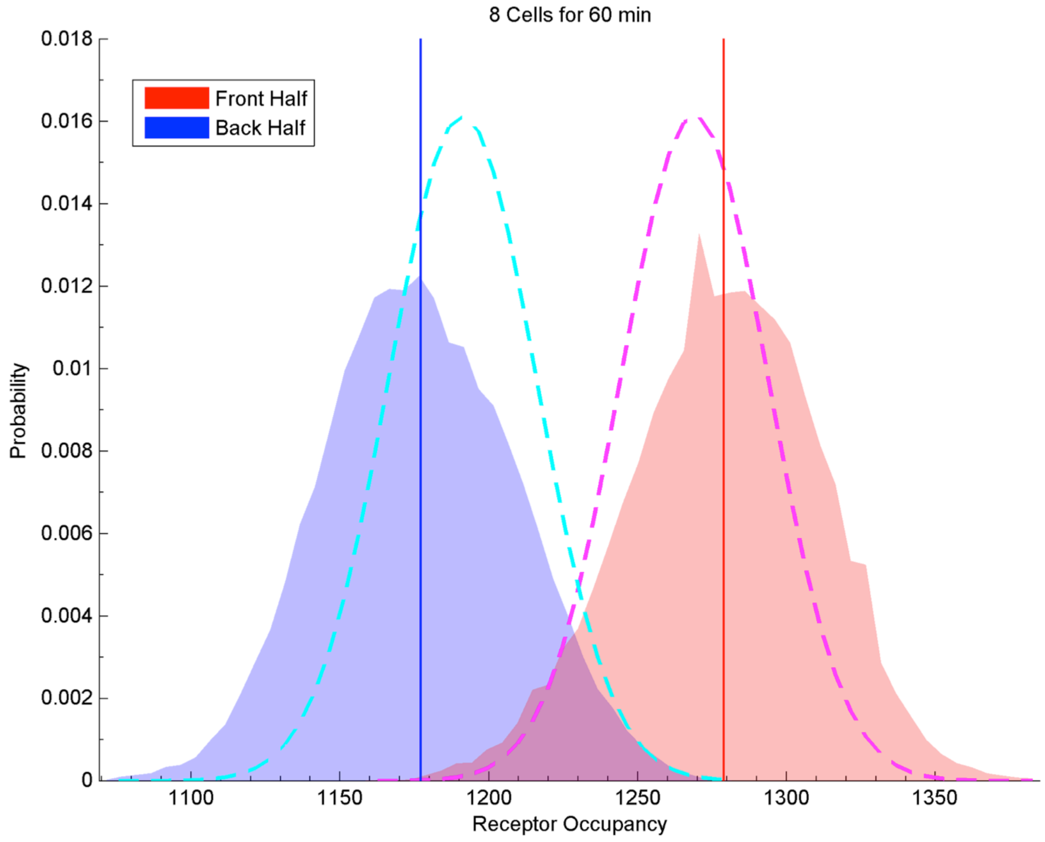

Figure E2: Front & Back Occupancy Distributions
This figure is similar to Fig 4C except that the radius of the cell is smaller (1.75µm) than the simulations presented in the main text (2.5µm). Note that the overlap region is significantly larger here than in Fig 4C.
