# Supplementary material for: Testing the limits of gradient sensing
Source: PLoS Comput Biol. 2017 Feb 16;13(2):e1005386. doi: 10.1371/journal.pcbi.1005386 (PMC5347372; doi:10.1371/journal.pcbi.1005386)
Supplement: S1 DataSet — This ZIP archive contains Matlab formatted data files and Matlab scripts (with instructions) needed to generate the curves shown in Figs 2 & 3. This archive also contains a DOCX file with more detailed information. See also ‘S5 DataSet’, ‘S6 DataSet’ and ‘S7 DataSet’. (ZIP) [file pcbi.1005386.s006.zip › ReadMe Figs 2 & 3.docx]

“Testing the Limits of Gradient Sensing” – VV Lakhani and TC Elston

PLoS Computational Biology 2017

[Timothy_Elston@med.unc.edu](mailto:Timothy_Elston@med.unc.edu)

[Vinal.Lakhani@gmail.com](mailto:Vinal.Lakhani@gmail.com)

To generate the figures shown in Fig 2 and 3, use the Matlab scripts provided. Before running the scripts, you will need to load one of the datasets provided here. In this text, we first describe what simulation datasets each Matlab formatted data file contains. Second, we describe the variable names and what values they contain.

**DataSet1.mat** – Simulation data used to generate *Figures 2, 3A – 3B*. Sixteen simulations of a cell in uniform pheromone concentration. The concentration is equal to the K_D_ of the receptor; the reaction rates are “fast”: k_on_ ~ 10^6^ (M · s)^-1^ and k_off_ ~ 10^-2^ 1/s. **DOWNLOAD this dataset from ‘S5 DataSet’**

**DataSet2.mat** – Simulation data used to generate the *blue curve in Figure 3C*. Twenty-six simulations of a cell in uniform pheromone concentration. The concentration is equal to the K_D_ of the receptor; the reaction rates are “slow”: k_on_ ~ 10^5^ (M · s)^-1^ and k_off_ ~ 10^-3^ 1/s. **DOWNLOAD this dataset from ‘S6 DataSet’ and ‘S7 DataSet’**

**DataSet3.mat** – Simulation data used to generate the *green curve in Figure 3C*. Eight simulations of a cell in uniform pheromone concentration. These are simulations of the ligand absorbing/endocytosis model. That is, the receptor cannot unbind/release ligand back into the environment; instead, a bound receptor is endocytosed. The concentration is the same as above; the reaction rates are “slow”: k_on_ ~ 10^5^ (M · s)^-1^ and k_endo_ ~ 10^-3^ 1/s.

**DataSet4.mat** – Simulation data used to generate the *blue curve in Figure 3D*. Ten simulations of a cell in uniform pheromone concentration. The concentration is equal to the K_D_ of the receptor; the reaction rates are artificially fast (500 times faster than reported in literature): k_on_ ~ 5×10^7^ (M · s)^-1^ and k_off_ ~ 0.5 1/s.

**DataSet5.mat** – Simulation data used to generate the *green curve in Figure 3D*. Ten simulations of a cell in uniform pheromone concentration. These are simulations of the ligand absorbing/endocytosis model. That is, the receptor cannot unbind/release ligand back into the environment; instead, a bound receptor is endocytosed. The concentration is the same as above; the reaction rates are artificially fast (500 times faster than reported in literature): k_on_ ~ 5×10^7^ (M · s)^-1^ and k_endo_ ~ 0.5 1/s.

Now, we briefly describe the variable names and what values they contain. The variables are listed alphabetically as they appear when the file is loaded in Matlab. In the description below “data” or “simulated data” refers to the number of occupied/bound receptors.

**Berezhkovskii** – CV^2^ values calculated using the Berezhkovskii & Szabo model (Eqn 5)

**CV2** – CV^2^ values from the simulated data

Colors – Stores a unique color for each simulation

D – Diffusion constant used in the simulated data

N – Total number of receptors simulated in each simulation

SaveName – Name of simulation data set

T – Total simulation time in seconds (s)

Tavg – Array denoting various lengths of Time-averaging (s). See also ‘nt’.

c – Target pheromone concentration (nM) at center of cell

devnt – For each length of Time-averaging, this variables holds the standard deviation from mean; calculated from simulated data (# Ste2*)

dt – size of time step (s) for recorded data. See ‘t’ and ‘noft’.

keb – Endocytosis Rate for a bound receptor (nM · s)^-1^

koff – Unbinding rate (1/s)

kon – Binding rate (nM · s)^-1^

nCells – number of simulations in this DataSet

neq – theoretical number of bound receptors at equilibrium

**noft** – A (T+1) × nCells matrix recording the number of bound receptors at each time point (row) for each simulation (column). The i^th^ row is at t = i * dt. Row 1 is at t=0s; row 2 is at t=1s etc.

**nt** – Time-averaged number of bound receptors. Like ‘noft’, the i^th^ row indicates the time point, the j^th^ column identifies which simulation. The k^th^ slice indicates which length of time-averaging. For k=1, a time-averaging of ‘Tavg’(1) = 10 seconds; for k=2 ‘Tavg’(2) = 20 seconds etc.

“nt(i,j,k)” provides the number of bound receptors at time “i*dt” averaged over the past “Tavg(k)” seconds for the j^th^ simulation.

r – radius of simulated cell (μm)

smplsz – “Sample Size”, for each length of time-averaging (see ‘Tavg’) the number of data points used to calculate the variance, standard deviation. These values are used to calculate the standard error.

t – time (s). Array of time points for data output; step size by ‘dt’ (s)

tauN – τ_N_ as calculated and described by the Berezhkovskii and Szabo model

theo_varn – theoretical variance as calculated by Lauffenburger (Eqn 1b).

varn – variance calculated from simulated data

varnt – contains the time-averaged variance for each length of time-averaging calculated from simulated data
