# Supplementary material for: Testing the limits of gradient sensing
Source: PLoS Comput Biol. 2017 Feb 16;13(2):e1005386. doi: 10.1371/journal.pcbi.1005386 (PMC5347372; doi:10.1371/journal.pcbi.1005386)
Supplement: S2 DataSet — This ZIP archive contains Matlab formatted data files and Matlab scripts (with instructions) needed to generate the curves shown in Figs 4, 5 & 8. This archive also contains a DOCX file with more detailed information. See also ‘S8 DataSet’, ‘S9 DataSet’, ‘S10 DataSet’, ‘S11 DataSet’ and ‘S12 DataSet’. (ZIP) [file pcbi.1005386.s007.zip › ReadMe Figs 4, 5 & 8.docx]

“Testing the Limits of Gradient Sensing” – VV Lakhani and TC Elston

PLoS Computational Biology 2017

[Timothy_Elston@med.unc.edu](mailto:Timothy_Elston@med.unc.edu)

[Vinal.Lakhani@gmail.com](mailto:Vinal.Lakhani@gmail.com)

To generate the figures shown in Figs 4 – 5, use the Matlab script provided. Before running the scripts, you will need to load one of the datasets provided here. In this text, we first describe what simulation datasets each Matlab formatted data file contains. Second, we describe the variable names and what values they contain. Additional information can be found in “ReadMe Figs 2 & 3.docx” found elsewhere in the Supporting Information.

**DataSet6.mat** – Simulation data (number of occupied receptors in front and back halves) used to generate *Figure 4C*. Eight simulations of a cell in a 0.5 nM/μm gradient pheromone concentration. The midpoint of the gradient has a concentration equal to the K_D_ of the receptor; the reaction rates are “fast”: k_on_ ~ 10^6^ (M · s)^-1^ and k_off_ ~ 10^-2^ 1/s.

**DataSet6_Grad.mat** – Data (pheromone counts in a cylindrical lattice per time point) from the same simulations as above. This data is used to generate *Figure 4A*. **DOWNLOAD from ‘S8 DataSet’**

**DataSet7.mat** – Simulation data (number of occupied receptors in front and back halves) used to generate Figure 4D. Eight simulations of a cell in a 0.5 nM/μm gradient pheromone concentration. The midpoint of the gradient has a concentration equal to the K_D_ of the receptor; the reaction rates are “fast”: k_on_ ~ 10^6^ (M · s)^-1^ and k_off_ ~ 10^-2^ 1/s. These simulations are unique in that they allow pheromone to diffuse through the “cell membrane”.

**DataSet7_Grad.mat** – Data (pheromone counts in a cylindrical lattice per time point) from the same simulations as above. This data is used to generate *Figure 4B*.

**DataSet8.mat** – Simulation data (number of occupied receptors in front and back halves) used to generate *Figure 5A*. Eight simulations of a cell in a 0.5 nM/μm gradient pheromone concentration. The midpoint of the gradient has a concentration equal to the K_D_ of the receptor; the reaction rates are “slow”: k_on_ ~ 10^5^ (M · s)^-1^ and k_off_ ~ 10^-3^ 1/s.

**DataSet9.mat** – Simulation data (number of occupied receptors in front and back halves) used to generate *Figure 5B*. Eight simulations of a cell in a 0.5 nM/μm gradient pheromone concentration. The midpoint of the gradient has a concentration equal to the K_D_ of the receptor; the reaction rates are “slow”: k_on_ ~ 10^5^ (M · s)^-1^ and k_off_ ~ 10^-3^ 1/s. These simulations are unique in that they do not allow Ste2 to diffuse at all.

**DataSet12_Grad.mat** – Simulation data (pheromone counts in a cylindrical lattice per time point) from simulations of “Gradient Method 2”. Neither receptors nor reactions are simulation; only pheromone diffusion. A spherical, reflective surface represents the impermeable cell membrane. A gradient of 0.1 nM/μm is simulated. This data is used to generate *Figure 8A*. **DOWNLOAD from ‘S9 DataSet’ and ‘S10 DataSet’**

**DataSet13_Grad.mat** – Simulation data (pheromone counts in a cylindrical lattice per time point) from simulations of “Gradient Method 2”. Neither receptors nor reactions are simulation; only pheromone diffusion. No spherical, reflective surface is simulated; pheromone are allowed to diffuse freely throughout the volume. A gradient of 0.1 nM/μm is simulated. This data is used to generate *Figure 8B*. **DOWNLOAD from ‘S11 DataSet’ and ‘S12 DataSet’**

Now, we briefly describe the variable names and what values they contain. The variables are listed alphabetically as they appear when the file is loaded in Matlab. Other variable descriptions are found in “ReadMe Figs 2 & 3.docx” found elsewhere in the Supporting Information.

**DataSet[6,7,12,13]_Grad.mat** files

allcurrCs – A 4-dimensional matrix containing the concentration for all bins at all times for all simulations. The row and column correspond to a bin in the cylindrical lattice. The 3^rd^ dimension indicates the time point. The 4^th^ dimension indicates which simulation.

ar – list of all edges along the radial component of the cylindrical lattice

conc – target pheromone concentration at midpoint of gradient (nM)

dom – domain size (in Cartesian) of simulation. Units in μm.

dr – step size of radial component of cylindrical lattice (μm)

dx – step size of the x-position component of the cylindrical lattice (μm)

ex – similar to ‘ar’: list of all edges along the x-component of the cylindrical lattice

grad – pheromone gradient simulated during simulation (nM/μm)

m – number of bins along the x-component of the cylindrical lattice

meanCs – average pheromone concentration at each bin in the cylindrical lattice averaged over all time and all simulations

n – number of bins along the radial component of the cylindrical lattic

stdCs – standard deviation of pheromone concentration at each bin in the cylindrical lattice averaged over all time and all simulations

stdNs – standard deviation of number of pheromone molecules at each bin in the cylindrical lattice averaged over all time and all simulations

theomeanCs – theoretical expected concentration at each bin in the cylindrical lattice

theostdCs – theoretical standard deviation of pheromone concentration at each bin in the cylindrical lattice

vols – volume (μm^3^) of each bin in the cylindrical lattice

**DataSet[6-9].mat** files

cb – average pheromone concentration in the back half. The midpoint of the gradient is at x=0 and the midpoint of the back half is at x = -(1/2)*r.

cf – average pheromone concentration in the front half.

emp_neqb – average number of occupied receptors in the back half of cell, averaged over all time and all cells

emp_neqf – same as ‘emp_neqb’ except for the front half

g – pheromone gradient simulated (nM/μm)

neqb – theoretical expected number of occupied receptors in the back half of the cell

neqf – same as ‘neqb’ except for front half

noftb – number of occupied receptors in the back half for a given time (row) and a given simulation (column)

noftf – same as ‘noftb’ except for front half

theo_varnb – theoretical variance of occupied receptors in the back half (Eqn 1)

theo_varnf – same as ‘theo_varnb’ except for front half
