# Supplementary material for: Testing the limits of gradient sensing
Source: PLoS Comput Biol. 2017 Feb 16;13(2):e1005386. doi: 10.1371/journal.pcbi.1005386 (PMC5347372; doi:10.1371/journal.pcbi.1005386)
Supplement: S3 DataSet — This ZIP archive contains Matlab formatted data files and Matlab scripts (with instructions) needed to generate the curves shown in Figs 6, 7 & 10. This archive also contains a DOCX file with more detailed information. (ZIP) [file pcbi.1005386.s008.zip › ReadMe Figs 6, 7 & 10.docx]

“Testing the Limits of Gradient Sensing” – VV Lakhani and TC Elston

PLoS Computational Biology 2017

[Timothy_Elston@med.unc.edu](mailto:Timothy_Elston@med.unc.edu)

[Vinal.Lakhani@gmail.com](mailto:Vinal.Lakhani@gmail.com)

To generate the figures shown in Figs 6, 7 & 10, use the Matlab script provided. Before running the scripts, you will need to load one of the datasets provided here. In this text, we first describe what simulation datasets each Matlab formatted data file contains. Second, we describe the variable names and what values they contain. Additional information can be found in “ReadMe Figs 2 & 3.docx” and “ReadMe Figs 4, 5 & 8.docx” found elsewhere in the Supporting Information.

**DataSet10.mat** – Simulation data (average position of occupied receptors) used to generate *Figures 6 & 7B*. Sixteen simulations of a cell in a 0.1 nM/μm gradient pheromone concentration. The target midpoint of the gradient has a concentration equal to the K_D_ of the receptor; the reaction rates are “fast”: k_on_ ~ 10^6^ (M · s)^-1^ and k_off_ ~ 10^-2^ 1/s.

**DataSet11.mat** – Simulation data (average position of occupied receptors) used to generate *Figure 7A*. Twenty-six simulations of a cell in a 0.1 nM/μm gradient pheromone concentration. The target midpoint of the gradient has a concentration equal to the K_D_ of the receptor; the reaction rates are “slow”: k_on_ ~ 10^5^ (M · s)^-1^ and k_off_ ~ 10^-3^ 1/s.

**DataSet18.mat** – Simulation data (average position of Ste2* & confidence) used to generate *blue curve in Figure 10A*. Twenty simulations of a cell in 0.1 nM/μm pheromone gradient. The target midpoint of the gradient has a concentration equal to receptor’s K_D_; the reaction rates are “slow”: k_on_ ~ 10^5^ (M · s)^-1^ and k_off_ ~ 10^-3^ 1/s.

**DataSet19.mat** – Simulation data (average position of Ste2* & confidence) used to generate *blue curve in Figure 10B*. Twenty simulations of a cell in 0.1 nM/μm pheromone gradient. The target midpoint of the gradient has a concentration equal to receptor’s K_D_; the reaction rates are “fast”: k_on_ ~ 10^6^ (M · s)^-1^ and k_off_ ~ 10^-2^ 1/s.

**DataSet20.mat** – Simulation data (average position of Ste2* & confidence) used to generate *red curve in Figure 10A*. Sixteen simulations of a cell in 0.1 nM/μm pheromone gradient; additionally, these simulations included the “Bar1 Model” as outlined in the Methods. The target midpoint of the gradient has a concentration equal to receptor’s K_D_; the reaction rates are “slow”: k_on_ ~ 10^5^ (M · s)^-1^ and k_off_ ~ 10^-3^ 1/s.

**DataSet21.mat** – Simulation data (average position of Ste2* & confidence) used to generate *red curve in Figure 10B*. Sixteen simulations of a cell in 0.1 nM/μm pheromone gradient; additionally, these simulations included the “Bar1 Model” as outlined in the Methods. The target midpoint of the gradient has a concentration equal to receptor’s K_D_; the reaction rates are “fast”: k_on_ ~ 10^6^ (M · s)^-1^ and k_off_ ~ 10^-2^ 1/s.

Now, we briefly describe the variable names and what values they contain. The variables are listed alphabetically as they appear when the file is loaded in Matlab. In the description below “data” or “simulated data” refers to the number of occupied/bound receptors. Other variable descriptions are found in “ReadMe Figs 2 & 3.docx” and “ReadMe Figs 4, 5 & 8.docx” found elsewhere in the Supporting Information.

**DataSet[10,11,18,19,20,21].mat** files

AvgC – Vectors of the average position of all occupied receptors (Ste2*) at a given time (row) for a given simulation (3^rd^ dimension index); each position (Cartesian vector) is recorded as [x, y, z]

AvgCDir – Unit vectors whose direction are equal to the average Ste2* position at a given time (row) for a given simulation (3^rd^ dimension index); each position (Cartesian vector) is recorded as [x, y, z]

AvgCMag – Magnitude of each average position (of all Ste2*) vector at a given time (row) for a given simulation (3^rd^ dimension index)

**DataSet[18,19,20,21].mat** files

oldGMetMag – Confidence (Eqn 9) values at a given time (row) for a given simulation (3^rd^ dimension index)
