# Supplementary material for: Testing the limits of gradient sensing
Source: PLoS Comput Biol. 2017 Feb 16;13(2):e1005386. doi: 10.1371/journal.pcbi.1005386 (PMC5347372; doi:10.1371/journal.pcbi.1005386)
Supplement: S4 DataSet — This ZIP archive contains Matlab formatted data files and Matlab scripts (with instructions) needed to generate the curves shown in Fig 9. This archive also contains a DOCX file with more detailed information. (ZIP) [file pcbi.1005386.s009.zip › ReadMe Fig 9.docx]

“Testing the Limits of Gradient Sensing” – VV Lakhani and TC Elston

PLoS Computational Biology 2017

[Timothy_Elston@med.unc.edu](mailto:Timothy_Elston@med.unc.edu)

[Vinal.Lakhani@gmail.com](mailto:Vinal.Lakhani@gmail.com)

To generate the figures shown in Fig 9, use the Matlab script provided. Before running the scripts, you will need to load one of the datasets provided here. In this text, we describe what simulation datasets each Matlab formatted data file contains. Additional information can be found in “ReadMe Figs 2 & 3.docx”, “**ReadMe Figs 4, 5 & 8.docx**” and “ReadMe Figs 6, 7 & 10.docx” found elsewhere in the Supporting Information.

**DataSet14.mat** – Simulation data (number of occupied receptors in front and back halves of cell) used to generate *Figures 9A*. Twenty simulations of a cell in a 0.1 nM/μm gradient pheromone concentration. The midpoint of the gradient has a concentration equal to the K_D_ of the receptor; the reaction rates are “slow”: k_on_ ~ 10^5^ (M · s)^-1^ and k_off_ ~ 10^-3^ 1/s. Initially there are no pheromone molecules in the simulation volume; pheromone molecules are flowed in according to “Gradient Method 2”.

**DataSet15.mat** – Simulation data (number of occupied receptors in front and back halves of cell) used to generate *Figures 9B*. Twenty simulations of a cell in a 0.1 nM/μm gradient pheromone concentration. The midpoint of the gradient has a concentration equal to the K_D_ of the receptor; the reaction rates are “fast”: k_on_ ~ 10^6^ (M · s)^-1^ and k_off_ ~ 10^-2^ 1/s. Initially there are no pheromone molecules in the simulation volume; pheromone molecules are flowed in according to “Gradient Method 2”.

**DataSet16.mat** – Simulation data (number of occupied receptors in front and back halves of cell) used to generate *Figures 9C*. Eight simulations of a cell in a 0.1 nM/μm gradient pheromone concentration. The midpoint of the gradient has a concentration equal to 10 times the K_D_ of the receptor (69nM); the reaction rates are “slow”: k_on_ ~ 10^5^ (M · s)^-1^ and k_off_ ~ 10^-3^ 1/s. Initially there are no pheromone molecules in the simulation volume; pheromone molecules are flowed in according to “Gradient Method 2”.

**DataSet17.mat** – Simulation data (number of occupied receptors in front and back halves of cell) used to generate *Figures 9D*. Eight simulations of a cell in a 0.1 nM/μm gradient pheromone concentration. The midpoint of the gradient has a concentration equal to 10 times the K_D_ of the receptor (69nM); the reaction rates are “fast”: k_on_ ~ 10^6^ (M · s)^-1^ and k_off_ ~ 10^-2^ 1/s. Initially there are no pheromone molecules in the simulation volume; pheromone molecules are flowed in according to “Gradient Method 2”.

The variable names and descriptions are found in “ReadMe Figs 2 & 3.docx”, “**ReadMe Figs 4, 5 & 8.docx**” and “ReadMe Figs 6, 7 & 10.docx” found elsewhere in the Supporting Information.
